# Oesophageal varices predict complications in compensated advanced non-alcoholic fatty liver disease

**DOI:** 10.1016/j.jhepr.2023.100809

**Published:** 2023-06-07

**Authors:** Grazia Pennisi, Marco Enea, Mauro Viganò, Filippo Schepis, Victor de Ledinghen, Annalisa Berzigotti, Vincent Wai-Sun Wong, Anna Ludovica Fracanzani, Giada Sebastiani, Carmen Lara-Romero, Elisabetta Bugianesi, Gianluca Svegliati-Baroni, Fabio Marra, Alessio Aghemo, Luca Valenti, Vincenza Calvaruso, Antonio Colecchia, Gabriele Di Maria, Claudia La Mantia, Huapeng Lin, Yuly P. Mendoza, Nicola Pugliese, Federico Ravaioli, Manuel Romero-Gomez, Dario Saltini, Antonio Craxì, Vito Di Marco, Calogero Cammà, Salvatore Petta

**Affiliations:** 1 Sezione di Gastroenterologia e Epatologia, PROMISE, Università di Palermo, Palermo, Italy; 2 Hepatology Unit, Ospedale San Giuseppe, University of Milan, Milan, Italy; 3 Division of Gastroenterology, Azienda Ospedaliero-Universitaria di Modena and University of Modena and Reggio Emilia, Modena, Italy; 4 Centre d’Investigation de la Fibrose Hépatique, INSERM U1053, Hôpital Haut-Lévêque, Bordeaux University Hospital, Pessac, France; 5 Department of Visceral Surgery and Medicine, Inselspital, Bern University Hospital, University of Bern, Bern, Switzerland; 6 Department of Medicine and Therapeutics, The Chinese University of Hong Kong, Hong Kong; 7 Department of Pathophysiology and Transplantation, Ca’ Granda IRCCS Foundation, Policlinico Hospital, University of Milan, Milan, Italy; 8 Division of Gastroenterology and Hepatology, McGill University Health Centre, Montreal, QC, Canada; 9 UCM Digestive Diseases, Virgen del Rocio University Hospital, Institute of Biomedicine of Seville (HUVR/CSIC/US), CIBEREHD, University of Seville, Ciberehd, Seville, Spain; 10 Division of Gastroenterology, Department of Medical Sciences, University of Torino, Torino, Italy; 11 Liver Injury and Transplant Unit, Università Politecnica delle Marche, Ancona, Italy; 12 Dipartimento di Medicina Sperimentale e Clinica, University of Florence, Florence, Italy; 13 Research Center DENOTHE, University of Florence, Florence, Italy; 14 Department of Biomedical Sciences, Humanitas University, Pieve Emanuele, Italy; 15 Division of Internal Medicine and Hepatology, Department of Gastroenterology, IRCCS Humanitas Research Hospital, Rozzano, Italy; 16 Department of Pathophysiology and Transplantation, Università degli Studi di Milano, Milan, Italy; 17 Precision Medicine and Biological Resource Center, Department of Transfusion Medicine, Fondazione IRCCS Ca’ Granda Ospedale Maggiore Policlinico IRCCS, Milan, Italy; 18 IRCCS Azienda Ospedaliero-Universitaria di Bologna, Bologna, Italy

**Keywords:** varices, NAFLD, cACLD, liver decompensation, Portal Vein Thrombosis, baveno, liver stiffness

## Abstract

**Background & Aims:**

We aimed to evaluate the impact of oesophageal varices (OV) and their evolution on the risk of complications of compensated advanced chronic liver disease (cACLD) caused by non-alcoholic fatty liver disease (NAFLD). We also assessed the accuracy of non-invasive scores for predicting the development of complications and for identifying patients at low risk of high-risk OV.

**Methods:**

We performed a retrospective assessment of 629 patients with NAFLD-related cACLD who had baseline and follow-up oesophagogastroduodenoscopy and clinical follow-up to record decompensation, portal vein thrombosis (PVT), and hepatocellular carcinoma.

**Results:**

Small and large OV were observed at baseline in 30 and 15.9% of patients, respectively. The 4-year incidence of OV from absence at baseline, and that of progression from small to large OV were 16.3 and 22.4%, respectively. Diabetes and a ≥5% increase in BMI were associated with OV progression. Multivariate Cox regression revealed that small (hazard ratio [HR] 2.24, 95% CI 1.47–3.41) and large (HR 3.86, 95% CI 2.34–6.39) OV were independently associated with decompensation. When considering OV status and trajectories, small (HR 2.65, 95% CI 1.39–5.05) and large (HR 4.90, 95% CI 2.49–9.63) OV at baseline and/or follow-up were independently associated with decompensation compared with the absence of OV at baseline and/or follow-up. The presence of either small (HR 2.8, 95% CI 1.16–6.74) or large (HR 5.29, 95% CI 1.96–14.2) OV was also independently associated with incident PVT.

**Conclusion:**

In NAFLD-related cACLD, the presence, severity, and evolution of OV stratify the risk of developing decompensation and PVT.

**Impact and implications:**

Portal hypertension is the main driver of liver decompensation in chronic liver diseases, and its non-invasive markers can help risk prediction. The presence, severity, and progression of oesophageal varices stratify the risk of complications of non-alcoholic fatty liver disease. Easily obtainable laboratory values and liver stiffness measurement can identify patients at low risk for whom endoscopy may be withheld, and can also stratify the risk of liver-related complications.

## Highlights

• The presence, severity, and progression of oesophageal varices stratify complication risk in patients with non-alcoholic fatty liver disease-related compensated advanced chronic liver disease.
• Changes in oesophageal varices status during follow-up improved the risk stratification of portal hypertension-related complications.
• Non-invasive surrogates of portal hypertension also significantly predicted a higher risk of liver decompensation.

## Graphical abstract

**Figure:**
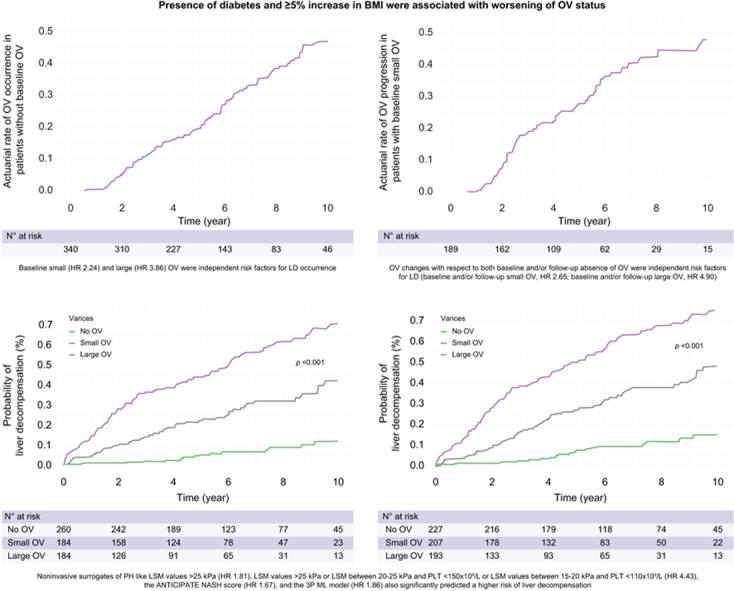

## Introduction

Non-alcoholic fatty liver disease (NAFLD) is an increasingly prevalent cause of compensated advanced chronic liver disease (cACLD) and its complications such as liver decompensation and hepatocellular carcinoma (HCC).^1^^,^^2^ Moreover, NAFLD also impacts direct and indirect costs for national health systems, the costs being higher for patients with liver-related complications.^3^

The development of portal hypertension (PH), and particularly of clinically significant portal hypertension (CSPH), defined as a hepatic venous pressure gradient ≥10 mmHg, is a key event to stratify disease severity and inform prognosis in patients with cACLD,^4^ including those with non-alcoholic steatohepatitis (NASH). The presence and grade of oesophageal varices (OV) further stratify the risk of decompensation in patients with viral or alcohol-related liver diseases.^5^^,^^6^ However, data regarding the clinical impact and natural history of OV in patients with NAFLD-related cirrhosis are limited.^7^^,^^8^

Simple clinical decision rules based on non-invasive methods have recently reduced the need for invasive tests (*i.e.* hepatic venous pressure gradient measurement and oesophagogastroduodenoscopy [OGD]) in the setting of cACLD.^9^ Specifically, the Baveno VII consensus^10^ recommended using the combination of liver stiffness measurement (LSM) ≤15 kPa and platelet count (PLT) ≥150 × 10^9^/L for ruling out CSPH, and using LSM ≥25 kPa alone, the combination of LSM between 20 and 25 kPa and PLT <150 × 10^9^/L, or the combination of LSM between 15 and 20 kPa and PLT <110 × 10^9^/L for ruling in CSPH, even though the latter seems to be suboptimal in obese patients with NAFLD. Moreover, in cACLD related to NASH, the ANTICIPATE NASH model (based on the combination of LSM, PLT, and BMI) and the machine learning three-parameter (3P ML) model (based on PLT, bilirubin, and international normalised ratio) have been proposed to predict CSPH but require further validation.^11^^,^^12^ According to the Baveno VII guidelines, non-invasive criteria (LSM <20 kPa and PLT >150 × 10^9^/L), already validated in the NAFLD setting, might be used to forego screening by OGD in patients with low likelihood of high-risk OV (<5%).^10^^,^^13^ The literature has also proposed criteria based on easily accessible clinical data such as PLT and albumin values, such as the Rete Sicilia Selezione Terapia (RESIST) criteria, in settings where LSM is not available.^14^

In the present study, we aimed to evaluate the predictive value of OV and their evolution on the risk of developing decompensation, portal vein thrombosis (PVT), and HCC in patients with cACLD caused by NAFLD. We also assessed the natural history and risk factors for OV progression/regression, and prognostic accuracy of non-invasive scores for predicting the development of complications and for identifying patients at low risk of high-risk OV.

## Patients and methods

### Patient selection

Patients were sequentially recruited at multiple participating centres from April 2001 to March 2021 at the first diagnosis of NAFLD-related cACLD. Inclusion criteria were NAFLD-related cACLD, presence of an OGD within 6 months of the diagnosis, and availability of OGD and clinical follow-up findings. A total of 629 patients were enrolled. Data were retrospectively reviewed and analysed.

NAFLD cACLD was diagnosed by histopathologic findings of F3–F4 fibrosis according to the Kleiner scoring system^15^ and/or by LSM >12 kPa.^16^ LSM was obtained by the FibroScan® device (Echosens, Paris, France) using an M and/or XL probe. If only one LSM result was available, it was included in the main analysis irrespective of probe type and BMI. When two LSM values were available (one with each probe), the main analysis included the M probe measurement for BMI <30 kg/m^2^ and the XL probe result for BMI ≥30 kg/m^2^.^17^ In patients without histologic examinations, the diagnosis of NAFLD required ultrasonographic detection of steatosis plus at least one criterion of the metabolic syndrome (obesity, diabetes, arterial hypertension, or dyslipidaemia). Other causes of liver disease were ruled out, including alcohol intake >20 g/day during the previous year (evaluated by patient interviews on the amount, frequency, and type, and confirmed by at least one family member); viral (HBsAg, anti-HCV, and anti-HIV negativity) and autoimmune hepatitis; hereditary haemochromatosis; and alpha1-antitrypsin deficiency. Patients with baseline advanced (Child–Pugh B or C) cirrhosis, HCC, liver transplantation, OV banding, PVT or splenic vein thrombosis, or splenectomy were excluded. The study was conducted in accordance with the principles of the Helsinki Declaration, and with local and national laws. Approval was obtained from the hospital internal review boards and their ethics committees. Informed consent was obtained from all participants.

### Patient evaluation

Clinical and anthropometric data, including BMI, the presence of arterial hypertension, and type 2 diabetes, and data regarding intake of statins, low-dose aspirin, selective beta-blockers, non-selective beta-blockers, and anticoagulants were collected both at enrolment and at the OGD showing changes in OV status or at the last OGD in cases without changes in OV status. On the same day, a 12-h overnight fasting blood sample was drawn to determine serum levels of aspartate aminotransferase, alanine aminotransferase, PLT, albumin, total bilirubin, international normalised ratio, total and HDL cholesterol, triglycerides, and plasma glucose concentration.

Transient elastography was performed with the FibroScan® medical device, using the M and/or XL probes within 6 months of OGD. In each centre, LSM was assessed after a ≥4-h fast by a trained operator who had previously performed at least 300 examinations of patients with chronic liver disease. Only patients with 10 valid measurements and with reliable results according to published criteria were enrolled.^18^

OGD was performed by experienced operators at each hospital, regardless of the Baveno criteria to avoid endoscopy. At endoscopy, high-risk OV that warrant primary prophylaxis to prevent bleeding were defined by medium or large size or the presence of high-risk stigmata (red wale marks and cherry red spots).^10^ In patients without baseline high-risk OV, OGD was repeated as recommended by clinical guidelines. Patients with baseline high-risk OV or with progression to medium or large OV were treated with non-selective beta-blockers or underwent elastic banding, and no further OGD follow up was performed. Prophylaxis was not initiated for patients with small (F1) varices without red wale marks.

Liver-related complications were recorded during the entire follow-up period and were defined as the development of decompensation (occurrence of ultrasonographically proven ascites and/or variceal haemorrhage and/or encephalopathy and/or jaundice), PVT, or HCC. In patients with ultrasonic findings suggesting PVT, the diagnosis was confirmed by computed tomography or contrast-enhanced liver ultrasound. Surveillance ultrasonography for HCC was conducted every 6 months in accordance with international guidelines.^19^ Patients who developed hepatic complications during follow-up were evaluated for available therapies and/or liver transplantation, as appropriate.^19^^,^^20^

### Statistical analysis

Continuous variables were summarised as means ± SD, and categorical variables as frequencies and percentages. Univariate and multivariate Cox models were used to assess risk factors for decompensation, incident PVT, and HCC, as well as for OV occurrence/progression and regression. Variables to be included in the multivariate model were chosen based on clinical relevance and on their significance in univariate analysis (*p* <0.10). Variables in the final models with a *p* value of <0.05 were considered statistically significant. Results were expressed as adjusted hazard ratios (HRs) and their 95% CIs. In models assessing the impact of follow-up changes in OV and in non-invasive scores for PH in predicting decompensation, these variables were considered as time-dependent covariates.

The Baveno VI (LSM <20 kPa and PLT >150 × 10^9^/L), expanded Baveno VI (LSM <25 kPa and PLT >110 × 10^9^/L), and RESIST (albumin >3.6 g/dl and PLT >120 × 10^9^/L) criteria were evaluated. Performance evaluations were made in terms of number and percentage of spared endoscopies, number and percentage of undetected OV, sensitivity, specificity, positive predictive value, and negative predictive value. Bonferroni test adjustment was used for multiple comparisons.

As the area under the receiving operating characteristic curve focuses only on the predictive accuracy of a model, not considering cases in which a false-negative result is more harmful than a false-positive result, we also performed a decision curve analysis (DCA) for identifying the threshold probabilities for which the use of non-invasive criteria translates into the maximum net benefit of detecting high-risk OV.[21], [22], [23], [24]

DCA-evaluated prediction models in comparison with default strategies of performing OGD in all or no patients enabled an assessment of overall yield of prediction rules. DCA estimates a ‘net benefit’ for each prediction rule, defined as follows:

Net benefit = sensitivity × prevalence - (1 - specificity) × (1 - prevalence) × *w*

where *w* is the odds of correct diagnosis (high-risk OV in this case) across different threshold probabilities. In this setting, net benefit represents a composite of the benefit gained by performing OGD for proven high-risk OV in patients classified as high risk according to non-invasive scores (true positive) and risk/discomfort incurred owing to OGD in those without high-risk OV but who were classified as high risk according to non-invasive scores (false positive). Threshold probability represents a theoretical risk level where the expected benefit of treatment is equal to the expected benefit of withholding treatment (*e.g*. the benefit of OGD equals the risk of not performing them). Thus, net benefit is assessed across a range of threshold probabilities to identify the best diagnostic strategy for different risk scenarios.

All data were analysed using RStudio (RStudio Inc., Boston, MA, USA). DCA was implemented in R using code derived from Zhang *et al.*^25^ In addition to the base packages in R, tidy verse, survival, survminer, boot, reshape2, and readxl packages were used.

## Results

### Patient characteristics

Baseline characteristics (overall and stratified for presence/absence of high-risk OV) are shown in Table 1. The mean age was 63.7 years, and 59.6% of patients were male. Sixty percent of patients were obese, and the prevalence of diabetes and arterial hypertension were 68.8 and 69.2%, respectively. The mean serum PLT and albumin values were 149.1 × 10^9^/L and 3.9 g/dl, respectively.

**Table 1:** **Baseline demographic, metabolic, laboratory, and instrumental features of patients with NAFLD-related cACLD**.

	NAFLD cACLD entire cohort (N = 629)	NAFLD cACLD without high-risk OV (n = 529)	NAFLD cACLD with high-risk OV (n = 100)	*p* value
Mean age (years)	63.7 ± 9.1	63.5 ± 9.3	64.5 ± 8.2	0.30
Male sex	59.6%	57.8%	69%	0.03
Mean BMI (kg/m^2^)	31.4 ± 5.5	31.5 ± 5.6	30.6 ± 4.7	0.12
Obesity	59.8%	60%	58.6%	0.79
AST (IU/L)	48.1 ± 25.9	48.1 ± 26.3	48.1 ± 23.9	0.98
ALT (IU/L)	48.7 ± 32.0	49.7 ± 33.2	43.3 ± 23.8	0.07
PLT ( × 10^9^/L)	149.1 ± 72.8	155.0 ± 71.3	117.8 ± 72.8	<0.001
PLT <110 × 10^9^/L	33.8%	29.3%	57.6%	<0.001
PLT <120 × 10^9^/L	40.4%	35.4%	66.6%	<0.001
PLT <150 × 10^9^/L	58.5%	54.3%	80.8%	<0.001
Albumin (g/L)	3.9 ± 0.4	4.0 ± 0.4	3.7 ± 0.5	<0.001
Albumin <3.6 g/L	19%	15.1%	40.2%	<0.001
Blood glucose (mg/dl)	129.8 ± 48.5	127.9 ± 46.0	139.8 ± 59.5	0.03
Total cholesterol (mg/dl)	168.9 ± 47.4	169.4 ± 48.1	166.4 ± 43.0	0.59
Triglycerides (mg/dl)	133.8± 80.7	136.3± 81.4	119.7± 75.1	0.10
Type 2 diabetes	68.8%	67.7%	75%	0.14
Arterial hypertension	69.2%	69.4%	68%	0.78
LSM (kPa)	25.9 ± 15.4	24.6 ± 14.1	32.6 ± 19.4	<0.001
LSM >20 kPa	57%	54.5%	69.4%	0.007
LSM ≥25 kPa	42.1%	38.6%	60%	<0.001
LSM ≤15 kPa plus PLT ≥150 × 10^9^/L	15.3%	17.4%	4.2%	0.001
LSM >25 kPa, LSM between 20 and 25 kPa plus PLT <150 × 10^9^/L, or LSM between 15 and 20 kPa plus PLT <110 × 10^9^/L	70.2%	65.9%	91.6%	<0.001
Baseline or follow-up anticoagulation therapy	8.5%	6.5%	19.7%	<0.001
Baseline or follow-up statin therapy	32.2%	37.4%	35%	0.81
Baseline or follow-up non-selective beta-blocker therapy	31%	21.1%	85.3%	<0.001
Baseline or follow-up selective beta-blocker therapy	22.5%	24.8%	9.7%	0.003
Baseline or follow-up aspirin therapy	27.4%	28.3%	21.9%	0.46
OV				
Presence	45.9%			
High-risk OV	15.9%			
Mean follow-up	75.6 ± 41.5	78.2 ± 41.1	61.6 ± 41.0	<0.001

Differences between continuous data were assessed using the Student *t* test or Mann–Whitney *U* test. Differences between categorical variables were assessed using the χ^2^ test.

Level of significance *p* <0.05.

ALT, alanine aminotransferase; AST, aspartate aminotransferase; cACLD, compensated advanced chronic liver disease; LSM, liver stiffness measurement; NAFLD, non-alcoholic fatty liver disease; OV, oesophageal varices; PLT, platelet count.

OV were present in 45.9% of cases, and high-risk OV in 15.9%. The composite of PLT ≥150 × 10^9^/L mmc and LSM ≤15 kPa was observed in 15.3% of patients, supporting the absence of CSPH,^10^ whereas 42.1% of patients had LSM ≥25 kPa diagnostic of CSPH.^10^ Moreover, 70.2% of patients had LSM >25 kPa, LSM between 20 and 25 kPa plus PLT <150 × 10^9^/L, or LSM between 15 and 20 kPa plus PLT <110 × 10^9^/L, suggestive of being at risk for CSPH.^10^

### Diagnostic accuracy of the Baveno VI, expanded Baveno VI, and RESIST criteria for high-risk OV

The main analysis was conducted to maximise data for each non-invasive criterion. A separate analysis was conducted in the subgroup of patients in whom all three criteria (Baveno VI, expanded Baveno VI, and RESIST) were available for each participant.

In the entire cohort, 23.1% of patients met the Baveno VI criteria to rule out high-risk OV, of whom 18% had any grade OV and 3.7% had high-risk OV at baseline (Table S1 and Fig. S1). A total of 43.8% of patients met the expanded Baveno VI criteria, of whom 20.3% had any grade OV and 7.1% had high-risk OV (Table S1 and Fig. S1). Finally, 50.1% of patients met the RESIST criteria, of whom 25.7% had any grade OV and 5% had high-risk OV (Table S1 and Fig. S1). Consistently, negative predictive values for the presence of needing treatment were 96.2, 92.8, and 94.9% for the Baveno VI, expanded Baveno VI, and RESIST criteria, respectively (Table S1). When looking at the group of patients in whom all the three non-invasive criteria were concomitantly available, similar results were observed (Table S1).

**Fig. 1:**
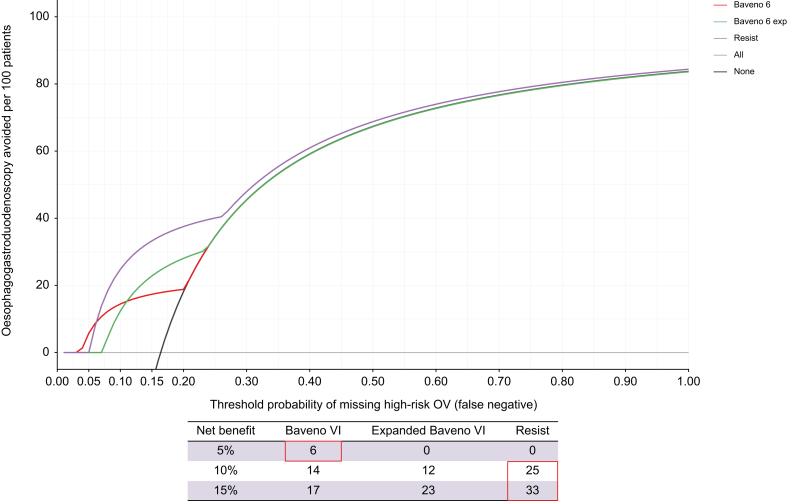
Net benefits by decision curve analyses of Baveno VI, expanded Baveno VI, and RESIST criteria for ruling out varices needing treatment. OV, oesophageal varices; RESIST, Rete Sicilia Selezione Terapia.

The net benefits of the Baveno VI, expanded Baveno VI, and RESIST criteria for ruling out high-risk OV are displayed in Fig. 1, showing the number of OGD avoided per 100 patients through a range of different threshold probabilities of missing high-risk OV. At the risk threshold of 5%, the net benefit was modest but highest for the Baveno VI criteria. At the risk thresholds higher than 10%, and 15% and higher, the RESIST criteria outperformed both the Baveno VI and expanded Baveno VI criteria.

### OV progression/regression in NAFLD cACLD

**Fig. 2:**
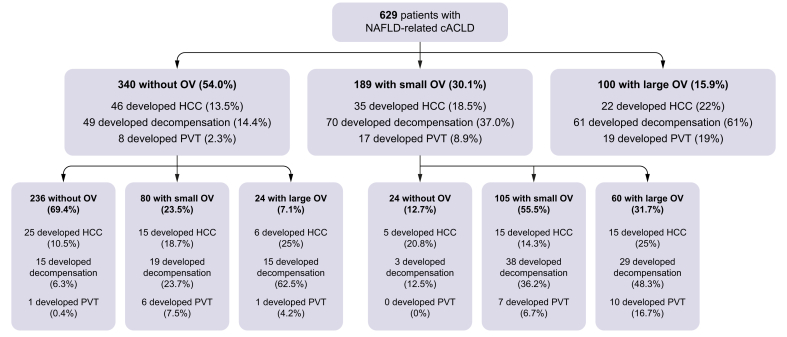
Crude rate of liver decompensation, portal thrombosis, and HCC occurrence according to baseline OV and follow-up changes in OV. cACLD, compensated advanced chronic liver disease; HCC, hepatocellular carcinoma; NAFLD, non-alcoholic fatty liver disease; OV, oesophageal varices; PVT, portal vein thrombosis.

Fig. 2 depicts the crude rate of OV progression in patients without or with baseline small OV and the crude rate of OV regression in patients with baseline small OV.

#### Occurrence of OV in patients without baseline OV

**Fig. 3:**
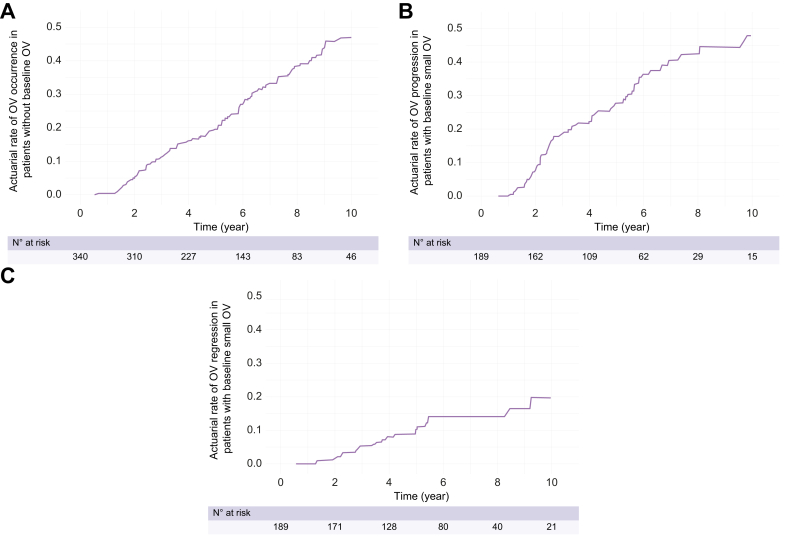
**Actuarial rates of OV evolution during follow-up**. (A) OV occurrence in patients without baseline OV, (B) OV progression in patients with baseline small OV, and (C) OV regression in patients with baseline small OV. Kaplan–Meier curves were also used to depict the time-dependent risk of developing events. OV, oesophageal varices.

The actuarial rate of OV development in patients without baseline OV was 16.3% at 4 years (Fig. 3A). Baseline diabetes was the only variable that predicted OV development (HR 1.75, 95% CI 1.09–2.81, *p* = 0.01) (Fig. S2A). No association was found between non-selective beta-blocker intake and OV development (*p* = 0.46) (Fig. S2B).

#### Progression of OV in patients with baseline small OV

The actuarial rate of OV progression in patients with baseline small OV was 22.4% at 4 years (Fig. 3B). Notably, 15% of patients experienced OV progression compared with those (13.9%) who have not used non-selective beta-blocker prophylaxis before the follow-up OGD. The progressive increasing of BMI (5, 7, and 10%), compared with baseline, predicted OV progression (delta BMI >5%, HR 2.28, 95% CI 1.19–4.36, *p* = 0.01; delta BMI >7%, HR 2.21, 95% CI 1.02–4.77, *p* = 0.04; delta BMI >10% HR 3.53, 95% CI 1.37–9.07, *p* = 0.008) (Fig. S3A–C). No other variables, including non-selective beta-blocker intake, predicted OV progression (*p* = 0.50) (Fig. S3D).

#### Regression of OV in patients with baseline small OV

The actuarial rate of OV regression in patients with baseline small OV was 8% at 4 years (Fig. 3C). No factors predicted OV regression, including lower rates of BMI increase (BMI increase of 5%, *p* = 0.18), and non-selective beta-blocker intake (*p* = 0.72).

### OV impact the risk of liver-related complications in NAFLD cACLD

During a mean follow-up of 75.6 months, 180 patients experienced decompensation, 103 developed HCC, and 44 developed PVT.

#### Liver decompensation

**Fig. 4:**
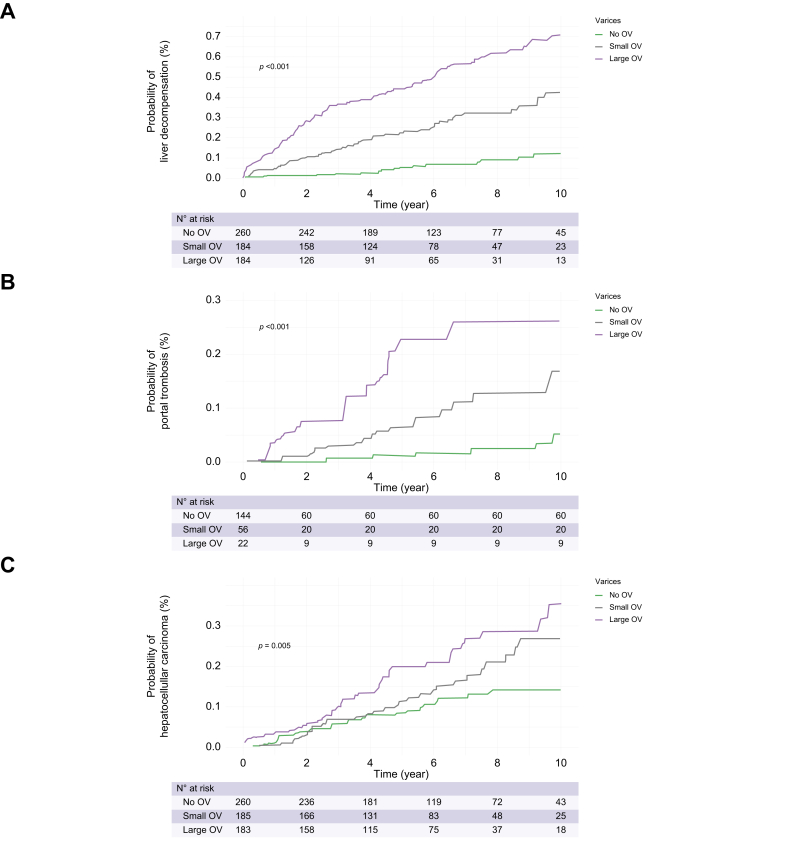
**Actuarial rates of liver-related events occurrence according to baselineabsent, small and large OV**. Actuarial rates of (A) liver decompensation, (B) portal thrombosis, and (C) hepatocellular carcinoma. Kaplan–Meier curves were also used to depict the time-dependent risk of developing events. Level of significance *p* <0.05. OV, oesophageal varices.

Fig. 2 shows the crude rate of decompensation at the end of follow-up according to OV status. Consistently, the Kaplan–Meier curve (Fig. 4A) showed that the actuarial rate of incident decompensation was lowest in patients without OV (1.8% at 1 year, 5.2% at 3 years, and 9.3% at 5 years), intermediate in those with small OV (4.3% at 1 year, 18.5% at 3 years, and 29.1% at 5 years), and highest in those with large OV (23.3% at 1 year, 46.4% at 3 years, and 50.7% at 5 years). Similarly, Fig. S4 depicts that the risk of decompensation was significantly higher in patients with LSM ≥25 kPa (considered as a non-invasive rule-in of CSPH) compared with their counterparts, significantly higher in patients with a 3P ML model >0.663 compared with their counterparts, and significantly lower in patients with LSM ≤15 kPa plus PLT ≥150 × 10^9^/L (considered as non-invasive rule-out of CSPH) compared with their counterparts. Congruently, patients at higher risk of CSPH because of LSM values >25 kPa, between 20 and 25 kPa and PLT <150 × 10^9^/L, or between 15 and 20 kPa and PLT <110 × 10^9^/L had a significantly higher risk of decompensation (Fig. S4). Finally, LSM alone (HR 1.03, 95% CI 1.02–1.04, *p* <0.001), the ANTICIPATE NASH score (HR 1.81, 95% CI 1.62–2.02, *p* <0.001), and the 3P ML model (HR 2.10, 95% CI 1.79–2.46, *p* <0.001) also significantly predicted a higher risk of decompensation. The univariate Cox regression models showed a significantly higher diagnostic accuracy for the prediction of decompensation by OV status, ANTICIPATE NASH score, and 3P ML model compared with all the other non-invasive scores (Table S2, top).

Multivariate Cox regression disclosed that the presence of small (HR 2.24, 95% CI 1.47–3.41, *p* <0.001) and large (HR 3.86, 95% CI 2.34–6.39, *p* <0.001) OV, PLT <150 × 10^9^/L (HR 3.41, 95% CI 2.11–5.52, *p* <0.001), age >65 years (HR 1.50, 95% CI 1.07–2.11, *p* = 0.01), albumin <3.6 g/L (HR 2.71, 95% CI 1.89–3.88, *p* <0.001), and therapy with non-selective beta-blockers (HR 1.56, 95% CI 1.04–2.35, *p* = 0.03) were independent risk factors for incident decompensation, whereas female sex (HR 0.48, 95% CI 0.33–0.70, *p* <0.001) had a protective role (Table 2). Replacement of OV with non-invasive markers of PH into the model confirmed the latter as independent predictors (HR 1.81, 95% CI 1.24–2.65, *p* = 0.002 for LSM ≥25 kPa; HR 4.43, 95% CI 2.12–9.27, *p* <0.001 for LSM values >25 kPa, LSM values between 20 and 25 kPa and PLT <150 × 10^9^/L, or LSM values between 15 and 20 kPa and PLT <110 × 10^9^/L; HR 1.67, 95% CI 1.47–1.90, *p* <0.001 for ANTICIPATE NASH score; HR 1.86, 95% CI 1.53–2.27, *p* <0.001 for 3P ML model) except for LSM ≤15 kPa plus PLT ≥150 × 10^9^/L (HR 0.56, 95% CI 0.29–1.07, *p* = 0.08). The generated Cox regression models showed a similar good diagnostic accuracy for the prediction of decompensation (Table S2, bottom).

**Table 2:** **Multivariate analysis of factors associated with liver decompensation (upper), HCC (middle), and portal thrombosis (bottom) in the entire cohort of patients with NAFLD-related cACLD**.

Variable	Hazard ratio (95% CI), *p* value					
**Liver decompensation**						
Female sex	0.48 (0.33–0.70), <0.001	0.45 (0.30–0.68), <0.001	0.41 (0.27–0.61), <0.001	0.44 (0.29–0.67), <0.001	0.46 (0.31–0.68), <0.001	0.50 (0.34–0.73), <0.001
Age ≥65 years	1.50 (1.07–2.11), 0.01	1.28 (0.89–1.85), 0.18	1.27 (0.89–1.82), 0.18	1.29 (0.90–1.86), 0.16	1.21 (0.84–1.75), 0.30	0.73 (0.52–1.03), 0.09
BMI ≥30 kg/m^2^	0.90 (0.65–1.27), 0.55	0.77 (0.54–1.11), 0.16	0.76 (0.53–1.08), 0.12	0.72 (0.50–1.03), 0.07	—	1.15 (0.82–1.60), 0.42
Small OV *vs*. no OV	2.24 (1.47–3.41), <0.001	—	—	—	—	
Large OV *vs.* no OV	3.86 (2.34–6.39), <0.001	—	—	—	—	
LSM ≥25 kPa		1.81 (1.24–2.65), 0.002				
LSM ≤15 kPa plus PLT ≥150 × 10^9^/L			0.56 (0.29–1.07), 0.08			
LSM >25 kPa, LSM between 20 and 25 kPa plus PLT <150 × 10^9^/L, or LSM between 15 and 20 kPa plus PLT <110 × 10^9^/L				4.43 (2.12–9.27), <0.001		
ARNTICIPATE NASH score					1.67 (1.47–1.90), <0.001	
3P ML model						1.86 (1.53–2.27), <0.001
PLT <150 × 10^9^/L	3.41 (2.11–5.52) <0.001	3.47 (2.08–5.79), <0.001	—	—	—	—
Albumin <3.6 g/dl	2.71 (1.89–3.88) <0.001	2.67 (1.82–3.92), <0.001	2.88 (1.98–4.18), <0.001	2.89 (1.98–4.42), <0.001	2.40 (1.62–3.56), <0.001	2.69 (1.86–3.89), <0.001
Statin therapy	0.76 (0.53–1.10), 0.14	0.63 (0.42–0.95), 0.02	0.81 (0.56–1.17), 0.25	0.61 (0.41–0.91), 0.01	0.69 (0.46–1.03), 0.07	0.80 (0.56–1.15), 0.23
Non-selective beta-blocker therapy	1.56 (1.04–2.35), 0.03	2.67 (1.84–3.89), <0.001	3.09 (2.15–4.43), <0.001	2.81 (1.93–4.08), <0.001	2.55 (1.75–3.72), <0.001	2.54 (1.79–3.60), <0.001

**HCC**						
Female Sex	0.27 (0.15–0.49), <0.001	0.21 (0.11–0.42), <0.001	0.28 (0.15–0.52), <0.001	0.21 (0.10–0.42), <0.001	0.20 (0.10–0.40), <0.001	0.27 (0.15–0.49), <0.001
Age ≥65 years	1.75 (1.08–2.84), 0.02	1.77 (1.04–2.99), 0.03	1.79 (1.07–3.00), 0.02	1.68 (1.0–2.84), 0.04	1.93 (1.16–3.22), 0.001	0.57 (0.35–1.42), 0.35
BMI ≥30 kg/m^2^	0.75 (0.47–1.21), 0.23	0.62 (0.36–1.04), 0.07	0.74 (0.45–1.23), 0.24	0.58 (0.34–1.01), 0.08	—	1.35 (0.84–2.17), 0.21
Small OV *vs*. no OV	1.21 (0.70–2.07), 0.49	—	—	—	—	
Large OV *vs*. no OV	1.17 (0.58–2.37), 0.66	—	—	—	—	
LSM ≥25 kPa		1.05 (0.62–1.78), 0.86		—	—	
LSM ≤15 kPa plus PLT ≥150 × 10^9^/L			0.65 (0.28–1.55), 0.33	—	—	
LSM >25 kPa, LSM between 20 and 25 kPa plus PLT <150 × 10^9^/L, or LSM between 15 and 20 kPa plus PLT <110 × 10^9^/L				1.87 (0.89–3.95), 0.09	—	
ANTICIPATE NASH score				—	1.15 (0.98–1.34), 0.09	
3P ML model						1.32 (1.01–1.73), 0.03
PLT <150 × 10^9^/L	2.18 (1.21–3.92), 0.009	1.98 (1.06–3.69), 0.03	—	—	—	—
Albumin <3.6 g/dl	1.67 (0.98–2.85), 0.06	2.28 (1.29–4.02), 0.004	1.60 (0.91–2.84), 0.10	2.26 (1.30–3.92), 0.003	2.10 (1.19–3.71), 0.01	2.36 (1.36–4.07), 0.002
Statin therapy	0.67 (0.40–1.13), 0.13	0.69 (0.40–1.21), 0.19	0.69 (0.40–1.18), 0.17	0.69 (0.40–1.20), 0.19	0.76 (0.44–1.32), 0.33	0.73 (0.43–1.22), 0.22
Non-selective beta-blocker therapy	1.67 (0.97–2.88), 0.07	1.74 (1.02–2.99), 0.03	1.88 (1.05–3.35), 0.03	1.77 (1.03–3.05), 0.03	1.76 (1.03–3.00), 0.03	1.74 (1.06–2.85), 0.02

**PVT**						
Female sex	0.42 (0.20–0.87), 0.02	0.20 (0.07–0.56), 0.002	0.51 (0.24–1.09), 0.08	0.21 (0.07–0.57), 0.002	0.21 (0.08–0.56), 0.002	0.48 (0.23–1.00), 0.05
Age ≥65 years	1.24 (0.64–2.42), 0.52	1.13 (0.52–2.44), 0.75	1.47 (0.71–3.01), 0.29	1.03 (0.49–2.20), 0.92	1.18 (0.56–2.48), 0.66	0.93 (0.48–1.79), 0.82
BMI ≥30 kg/m^2^	1.04 (0.54–1.99), 0.90	0.73 (0.35–1.53), 0.40	1.13 (0.56–2.31), 0.72	0.60 (0.29–1.25), 0.17	—	0.93 (0.49–1.76), 0.81
Small OV *vs*. no OV	2.80 (1.16–6.74), 0.02			—	—	—
Large OV *vs*. no OV	5.29 (1.96–14.2), 0.001			—	—	—
LSM ≥25 kPa		0.96 (0.45–2.04), 0.90		—	—	—
LSM ≤15 KPa plus PLT ≥150 × 10^9^/L			1.07 (0.31–3.75), 0.91	—	—	—
LSM>25 kPa, LSM between 20 and 25 kPa plus PLT <150 × 10^9^/L, or LSM between 15 and 20 kPa plus PLT <110 × 10^9^/L				3.86 (0.83–17.9), 0.08	—	—
ANTICIPATE NASH score				—	1.39 (1.06–1.81), 0.01	—
3P ML model						2.35 (1.54–3.58), <0.001
PLT <150 × 10^9^/L	4.48 (1.56–12.8), 0.005	5.00 (1.43–17.4), 0.01	—	—	—	—
Albumin <3.6 g/dl	1.79 (0.89–3.62), 0.10	1.76 (0.79–3.92), 0.16	2.46 (1.18–5.13), 0.01	1.78 (0.82–3.86), 0.14	1.46 (0.65–3.29), 0.36	1.89 (0.94–3.81), 0.07
Statin therapy	0.52 (0.24–1.13), 0.09	0.33 (0.12–0.87), 0.02	0.45 (0.20–1.03), 0.05	0.34 (0.13–0.89, 0.02	0.42 (0.16–1.10, 0.07	0.55 (0.25–1.20, 0.13
Non-selective beta-blocker therapy	1.56 (0.70–3.47), 0.27	5.57 (2.38–13.0), <0.001	1.31 (0.56–3.08), 0.52	5.64 (2.41–13.1) <0.001	5.28 (2.24–12.4), 0.001	2.50 (1.25–5.00), 0.009

Multivariate Cox models were used to assess risk factors for decompensation, incident PVT, and HCC.

Level of significance *p* <0.05.

3P ML, machine learning three-parameter; cACLD, compensated advanced chronic liver disease; HCC, hepatocellular carcinoma; LSM, liver stiffness measurement; NAFLD, non-alcoholic fatty liver disease; NASH, non-alcoholic steatohepatitis; OV, oesophageal varices; PLT, platelet count; PVT, portal vein thrombosis.

Subgroup analyses performed in patients stratified according to non-invasive markers of PH confirmed the independent association of OV with incident decompensation in almost all subgroups (Table S3).

**Fig. 5:**
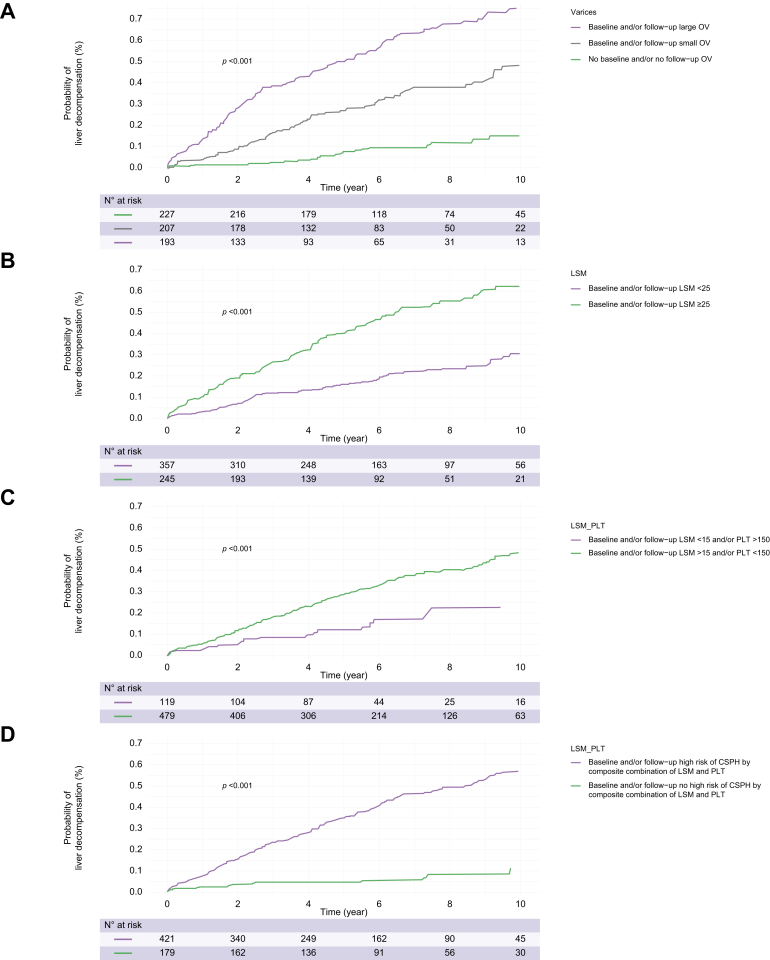
**Actuarial rates of liver decompensation according to follow-up changes in OV and in noninvasive markers of portal hypertension**. (A) follow-up changes in OV; (B) LSM ≥25 kPa; (C) LSM ≤15 kPa plus PLT ≥150 × 10^9^/L; and (D) LSM >25 kPa, LSM between 20 and 25 kPa and PLT <150 × 10^9^/L, or LSM between 15 and 20 kPa and PLT <110 × 10^9^/L status. Kaplan–Meier curves were also used to depict the time-dependent risk of developing events. Level of significance *p* <0.05. CSPH, clinically significant portal hypertension; LSM, liver stiffness measurement; OV, oesophageal varices; PLT, platelet count.

The availability of data about OV progression/regression during follow-up allowed us to assess the impact of their changes during follow-up on the risk of decompensation, and we considered OV changes that occurred before liver decompensation. Fig. 5A and Table S4 depict the actuarial risk of decompensation stratified according to OV status during follow-up. Specifically, we further considered three different classes: (1) patients without baseline and/or follow-up OV; (2) patients with baseline and/or follow-up small OV; and (3) patients with baseline and/or follow-up large OV. We also assessed the actuarial risk of decompensation stratified by changes in the status from baseline to follow-up of non-invasive markers of PH (Fig. 5B–D and Table S4). The generated univariate Cox regression models showed higher diagnostic accuracy for the prediction of decompensation by OV status with respect to non-invasive markers of PH (Table S5, top).

At multivariate Cox regression analysis, OV changes with respect to baseline and/or follow-up absence of OV were confirmed as independent risk factors for decompensation (baseline and/or follow-up small OV, HR 2.65, 95% CI 1.39–5.05, *p* = 0.002; baseline and/or follow-up large OV, HR 4.90, 95% CI 2.49–9.63, *p* <0.001) (Table 3). Replacing OV changes with variations in the status of non-invasive markers of PH into the model confirmed the latter as independent predictors of decompensation (Table 3). The generated Cox regression models showed a similar good diagnostic accuracy for the prediction of decompensation (Table S5, bottom).

**Table 3:** **Multivariate analysis of factors associated with liver decompensation by considering changes during follow-up of OV status or of non-invasive markers of portal hypertension in patients with NAFLD-related cACLD**.

Variable	Hazard ratio	95% CI	*p* value
**Model 1**			
Female sex	0.49	(0.33–0.72)	<0.001
Age ≥65 years	1.59	(1.11–2.28)	0.01
BMI ≥30 kg/m^2^	0.72	(0.51–1.03)	0.07
Baseline and/or follow-up small OV *vs*. baseline and/or follow-up no OV	2.65	(1.39–5.05)	0.002
Baseline and/or follow-up large OV *vs*. baseline and/or follow-up no OV	4.90	(2.49–9.63)	<0.001
PLT <150 × 10^9^/L	3.06	(1.86–5.03)	<0.001
Albumin <3.6 g/dl	2.52	(1.76–3.61)	<0.001
Statin therapy	0.68	(0.47–0.99)	0.04
Non-selective beta-blocker therapy	1.4	(0.96–2.05)	0.08
**Model 2**			
Female sex	0.49	(0.33–0.73)	<0.001
Age ≥65 years	1.42	(0.98–2.05)	0.06
BMI ≥30 kg/m^2^	0.76	(0.49–1.18)	0.22
Baseline and/or follow-up LSM ≥25 kPa *vs*. baseline and/or follow-up LSM <25 kPa	2.35	(1.59–3.47)	<0.001
PLT <150 × 10^9^/L	3.48	(2.07–5.85)	<0.001
Albumin <3.6 g/dl	2.49	(1.73–3.59)	<0.001
Statin therapy	0.61	(0.41–0.89)	0.01
Non-selective beta-blocker therapy	2.31	(1.56–3.42)	<0.001
**Model 3**			
Female sex	0.43	(0.28–0.67)	<0.001
Age ≥65 years	1.46	(0.98–2.17)	0.06
BMI ≥30 kg/m^2^	0.70	(0.48–1.03)	0.07
Baseline and/or follow-up LSM >15 kPa and/or PLT <150 × 10^9^/L *vs*. baseline and follow-up LSM ≤15 kPa plus PLT ≥150 × 10^9^/L	0.50	(0.29–0.89)	0.01
Albumin <3.6 g/dl	2.60	(1.78–3.80)	<0.001
Statin therapy	0.80	(0.55–1.17)	0.24
Non-selective beta-blocker therapy	2.78	(1.92–4.03)	<0.001
**Model 4**			
Female sex	0.44	(0.29–0.66)	<0.001
Age ≥65 years	1.32	(0.92–1.90)	0.13
BMI ≥30 kg/m^2^	0.71	(0.46–1.10)	0.12
Baseline and/or follow-up LSM >25 kPa, LSM between 20 and 25 kPa plus PLT <150 × 10^9^/L, or LSM between 15 and 20 kPa plus PLT <110 × 10^9^/L *vs*. baseline and/or follow-up LSM between 20 and 25 kPa plus PLT >150 × 10^9^/L, LSM between 15 and 20 kPa plus PLT >110 × 10^9^/L, or LSM <15 kPa	6.92	(2.99–16.01)	<0.0001
Albumin <3.6 g/dl	2.86	(2.01–4.05)	<0.001
Statin therapy	0.66	(0.46–0.96)	0.02
Non-selective beta-blocker therapy	2.52	(1.75–3.63)	<0.001

Multivariate Cox models were used to assess risk factors for decompensation.

Level of significance *p* <0.05.

cACLD, compensated advanced chronic liver disease; LSM, liver stiffness measurement; NAFLD, non-alcoholic fatty liver disease; OV, oesophageal varices; PLT, platelet count.

#### Portal vein thrombosis

Fig. 2 shows the crude rate of PVT at the end of follow-up according to OV status. Consistently, the Kaplan–Meier curve (Fig. 4B) showed that the actuarial rate of PVT incidence was lowest in patients without OV (0% at 1 year, 0.3% at 3 years, and 0.7% at 5 years), intermediate in those with small OV (0.5% at 1 year, 2.9% at 3 years, and 6.5% at 5 years), and highest in those with large OV (3.1% at 1 year, 7.6% at 3 years, and 22.7% at 5 years).

Multivariate Cox regression disclosed that the presence of small OV (HR 2.80, 95% CI 1.16–6.474, *p* = 0.02) and large OV (HR 5.29, 95% CI 1.96–14.2, *p* = 0.001), and PLT <150 × 10^9^/L (HR 4.48, 95% CI 1.56–12.8, *p* = 0.005) were independent risk factors for incident PVT, whereas female sex (HR 0.42, 95% CI 0.20–0.87, *p* = 0.02) was protective (Table 2). Replacement of OV with non-invasive markers of PH into the model disclosed that only the ANTICIPATE NASH score (HR 1.39, 95% CI 1.06–1.81, *p* = 0.01) and the 3P ML model (HR 2.35, 95% CI 1.54–3.58, *p* <0.001) were independently associated with incident PVT.

#### Hepatocellular carcinoma

Fig. 1 shows the crude rate of HCC at the end of follow-up according to OV status. Consistent with the crude rate, the Kaplan–Meier curve (Fig. 4C) showed that the actuarial rate of HCC incidence was lowest in patients without OV (0.9% at 1 year, 6.4% at 3 years, and 10% at 5 years), intermediate in those with small OV (2.1% at 1 year, 8.1% at 3 years, and 13.4% at 5 years), and highest in those with large OV (3% at 1 year, 9.5% at 3 years, and 21.7% at 5 years).

Multivariate Cox regression revealed that neither small (HR 1.21, 95% CI 0.70–2.07, *p* = 0.49) nor large (HR 1.17, 95% CI 0.58–2.37, *p* = 0.66) OV were associated with incident HCC, whereas age >65 years (HR 1.75, 95% CI 1.08–2.84, *p* = 0.02) and PLT<150 × 10^9^/L (HR 2.18, 95% CI 1.21–3.92, *p* = 0.009) were independent risk factors. Female sex had a protective effect (HR 0.27, 95% CI 0.15–0.49, *p* <0.001) (Table 2). Replacing OV with non-invasive markers of PH into the model revealed that only the 3P ML model (HR 1.32, 95% CI 1.01–1.73, *p* = 0.03) was independently associated with incident HCC (Table 2).

## Discussion

In this large retrospective multicentre cohort study of patients with NAFLD-related cACLD, we found that the presence and size of OV, as signs of CSPH, independently predicted the risk of developing decompensation and PVT. We also showed that changes of OV status during follow-up, primarily related to metabolic risk factors such as adiposity and diabetes, improved the risk stratification of PH-related complications.

PH significantly affects the natural history of cACLD,^4^^,^^5^ but the measurement of the hepatic venous pressure gradient is not often used in clinical practice because of its limited availability, costs, and invasiveness. Consequently, surrogate markers of CSPH such as OV are expected to facilitate the risk stratification of liver-related complications in patients with cACLD, including those with NAFLD.

In the present study, we found that among patients with NAFLD with cACLD, the 5-year risk of developing decompensation increased from 9% in patients without OV to 29% in those with small and further to 50% in those with large baseline OV. Notably, after adjusting for well-known clinical risk factors, we confirmed that the baseline presence of small and large OV, observed in 30 and 15.9% of patients, respectively, increased the risk of decompensation by twofold and nearly fourfold, respectively. Our data confirm what is already described in patients with cirrhosis caused by mixed aetiologies or HCV infection.^5^^,^^6^ Moreover, a limited number of smaller studies have also identified the presence of OV as an independent risk factor for decompensation in patients with NAFLD cirrhosis.^7^^,^^8^ However, these studies did not discriminate between small and large OV, limiting the clinical translatability of their results.

We also investigated the impact of time-dependent OV evolution on the risk of developing decompensation. To our knowledge, we report the first demonstration that, after adjusting for confounders, the risk of decompensation was 2.5- and 5-fold higher in patients with small and large baseline and/or follow-up large OV, respectively, compared with those without baseline and/or follow-up OV. Overall, we showed that in the setting of NAFLD cACLD, OV assessment, as a surrogate marker of CSPH, can stratify the risk of decompensation.

We observed that all of the Baveno VII non-invasive markers of PH considered separately, the ANTICIPATE NASH score, and the new proposed 3P ML model^12^ can identify patients at higher or lower risk of decompensation. Moreover, when assessing time-dependent changes of these variables, baseline and/or follow-up presence of non-invasive markers of PH identified patients at higher risk of developing decompensation. Our data raise the question of whether non-invasive tools can replace the invasive evaluation of OV to predict decompensation. Our findings suggest that OV assessment and the use of the ANTICIPATE NASH score or the 3P ML model are more accurate in the prediction of decompensation than are other non-invasive clinical prediction rules proposed by the Baveno VII consensus to rule in and rule out CSPH. We also demonstrated that the presence and severity of OV accurately stratified the risk of decompensation in the subgroup of patients stratified at high or low risk of PH by non-invasive scores. All in all, these data, while demonstrating the clinical utility of OV evaluation, suggest that the use of non-invasive scores and comprehensive clinical and biochemical evaluation can be acceptable, especially in low-resource settings, and that the ANTICIPATE NASH score and the 3P ML model showed higher accuracy. Moreover, the clinical relevance of our data needs to be confirmed prospectively in patients with cACLD and suspected CSPH taking non-selective beta-blockers to reduce the risk of decompensation as recommended by the Baveno VII consensus.^10^

We also confirmed male sex, older age, and lower PLT and albumin levels as further risk factors for decompensation. Treatment with non-selective beta-blockers was also associated with a higher risk of decompensation, even when this association was lost when included into the model changes in OV status. In our setting, treatment with non-selective beta-blockers appears as a marker of more severe disease, that is, large OV, or the development of large OV. Further *ad hoc* studies are needed to explore in real life the protective effects of this class of drugs against decompensation as suggested by the PREDESCI trial in patients with CSPH and no or small OV.^26^

Non-neoplastic PVT is another complication of cACLD and is primarily related to PH. To our knowledge, our study is the first reported demonstration that in the setting of NAFLD cACLD, the presence and severity of OV accurately stratify the risk of PVT. The 5-year risk was negligible in patients without OV (0.7%), low in those with small OV (6.5%), and high in those with large OV (22.7%). Notably, after adjusting for confounders, the presence of small and large OV increased the risk of PVT threefold and fivefold, respectively. A prior study of over 1,000 patients with cirrhosis identified OV as a risk factor for PVT but was focused on patients with cirrhosis of aetiologies other than NAFLD and did not discriminate between small and large OV.^27^ Our study also showed that the ANTICIPATE NASH score and the 3P ML model that use non-invasive markers of PH independently predicted incident PVT. All in all, these results suggest that PVT is directly related to the severity of PH.

Another relevant issue from our study lies in the description of the natural history and risk factors of OV evolution. Our observed 4-year incidence rates of OV in patients without baseline OV of 16.3% and of OV progression in patients with small OV at baseline of 22.4% are congruent with those of patients with cirrhosis of other aetiologies, even though studies on patients with HCV or alcohol-related cirrhosis reported higher rates of OV occurrence.[28], [29], [30], [31], [32] Notably, our identification of baseline diabetes and BMI increases of at least 5% as the main risk factors of OV development/progression highlight the clinical relevance of metabolic risk factors as drivers of PH-related complications in patients with NAFLD cACLD,^33^^,^^34^ and open new opportunities regarding the potential benefit of drugs to control diabetes and reduce body weight reduction in the management of PH. We also observed that 8% of patients with small OV at baseline experienced OV regression at 4-year follow-up, although no significant predictors were identified. Larger studies that also evaluate the potential regression of large OV are needed to elucidate this topic. Such studies could evaluate the rate and risk factors for the regression of PH and its clinical surrogates such as OV.

Finally, our evaluation of the non-invasive identification of patients at low risk of high-risk OV not requiring OGD screening confirmed the superiority of the Baveno VI criteria to the expanded Baveno criteria in terms of missing high-risk OV, although at the cost of a lower proportion of spared OGD.^13^ We also showed that the RESIST criteria can yield an acceptable accuracy in settings with limited access to LSM. In our DCA, the Baveno VI criteria showed a modest but higher net benefit than other scores for ruling out high-risk OV at a threshold probability of 5% of missing high-risk OV. In comparison, RESIST outperformed both the Baveno VI and expanded Baveno VI criteria at threshold probabilities of 10 and 15%. However, all scores missed small OV at threshold probabilities from 18 to 25%. This issue is clinically relevant because we clearly associated small OV with a higher risk of PH-related complications.

This study has several limitations that should be mentioned. The first is its retrospective design. Inclusion criteria that required a baseline OGD may have introduced a selection bias. We could not exclude surreptitious alcohol use during follow-up that could have affected OV progression. Although OV size was determined by several endoscopists, we did not test interobserver agreement. In addition, the lack of data regarding gastric varices and portal hypertensive gastropathy may affect the interpretation of our findings. Secondly, interobserver concordance of LSM examinations was not assessed, which may potentially limit the strength of our results. However, all tests were performed by expert operators following the same protocol and fulfilling validity criteria. Moreover, other studies assessing NAFLD by using FibroScan® evaluated multicentre cohorts tested by multiple operators.^35^^,^^36^ Finally, many studies have reported good interobserver concordance for LSM.^37^^,^^38^ The maximum 6-month interval between LSM and OGD could further bias our results. An additional methodological issue is the potentially limited external validity of the results for different populations and settings. We evaluated a cohort of patients with NAFLD-related compensated cirrhosis who were often obese and were referred to tertiary centres for liver disease and underwent OGD regardless of Baveno VI screening recommendations, limiting the applicability of the results in different populations, and especially in lean and Asiatic populations that were largely under-represented in this study. Finally, the lack of a competing risk analysis considering mortality and liver transplantation may affect the overall interpretation of our results.

In conclusion, we demonstrated the clinical impact of OV presence, severity, and evolution on the risks of decompensation and PVT in patients with cACLD caused by NAFLD and also revealed the role of metabolic risk factors as drivers of OV occurrence and progression.

## Financial support

The research leading to the results of this study has received funding from MIUR under PNRR M4C2I1.3 Heal Italia project PE00000019 CUP B73C22001250006 to SP. The project is supported by the Italian PNRR-MAD-2022-12375656 project. The project is supported by the Italian RF-2021-12372399 project. GS is supported by a Senior Salary Award from *Fonds de Recherche du Quebec – Sante* (#296306).

## Authors’ contributions

Designed the study, contributed to data acquisition, was responsible for writing the manuscript, and participated in statistical analysis: SP.

Responsible for the project and writing of the manuscript: GP, ME, MV, FS, VdL, AB, VWW, ALF, GS, MR-G, EB, GS-B, FM, AA, LV, VC, AC, GDM, CLM, HL, YM, NP, FR, CL-R, DS, AC, VDM, CC.

Seen and approved the final version of the manuscript: all authors.

## Data availability statement

Research data are confidential.

## Conflict of interest

The authors declare no conflicts of interest that pertain to this work.

Please refer to the accompanying ICMJE disclosure forms for further details.
