# Supplementary material for: Oesophageal varices predict complications in compensated advanced non-alcoholic fatty liver disease
Source: JHEP Rep. 2023 Jun 7;5(9):100809. doi: 10.1016/j.jhepr.2023.100809 (PMC10393808; doi:10.1016/j.jhepr.2023.100809)
Supplement: Multimedia component 1 [file mmc1.pdf]

# Oespohageal varices predict complications in compensated advanced non-alcoholic fatty liver disease

Grazia Pennisi, Marco Enea, Mauro Viganò, Filippo Schepis, Victor de Ledinghen, Annalisa Berzigotti, Vincent Wai-Sun Wong, Anna Ludovica Fracanzani, Giada Sebastiani, Carmen Lara-Romero, Elisabetta Bugianesi, Gianluca Svegliati-Baroni, Fabio Marra, Alessio Aghemo, Luca Valenti, Vincenza Calvaruso, Antonio Colecchia, Gabriele Di Maria, Claudia La Mantia, Huapeng Lin, Yuly P. Mendoza, Nicola Pugliese, Federico Ravaioli, Manuel Romero-Gomez, Dario Saltini, Antonio Craxì, Vito Di Marco, Calogero Cammà, Salvatore Petta

Table of contents

Contributors....................................................................................................2

Fig. S1....................................................................................................3

Fig. S2....................................................................................................4

Fig. S3....................................................................................................6

Fig. S4....................................................................................................10

Table S1....................................................................................................13

Table S2....................................................................................................15

Table S3....................................................................................................17

Table S4....................................................................................................18

Table S5....................................................................................................20

**Contributors**

S. Petta designed the study, contributed to data acquisition, was responsible for writing the manuscript, and participated in statistical analysis. G Pennisi, M Enea, M Viganò, F Schepis, V de Ledinghen, A Berzigotti, VW Wong, AL Fracanzani, G Sebastiani, M Romero-Gomez, E Bugianesi, G Svegliati-Baroni, F Marra, A Aghemo, L Valenti, V Calvaruso, A Colecchia, G Di Maria, C La Mantia, H Lin, Y Mendoza, N Pugliese, F Ravaioli, C Lara-Romero, D Saltini, A Craxì, V Di Marco, C Cammà were responsible for the project and writing of the manuscript. All authors have seen and approved the final version of the manuscript.

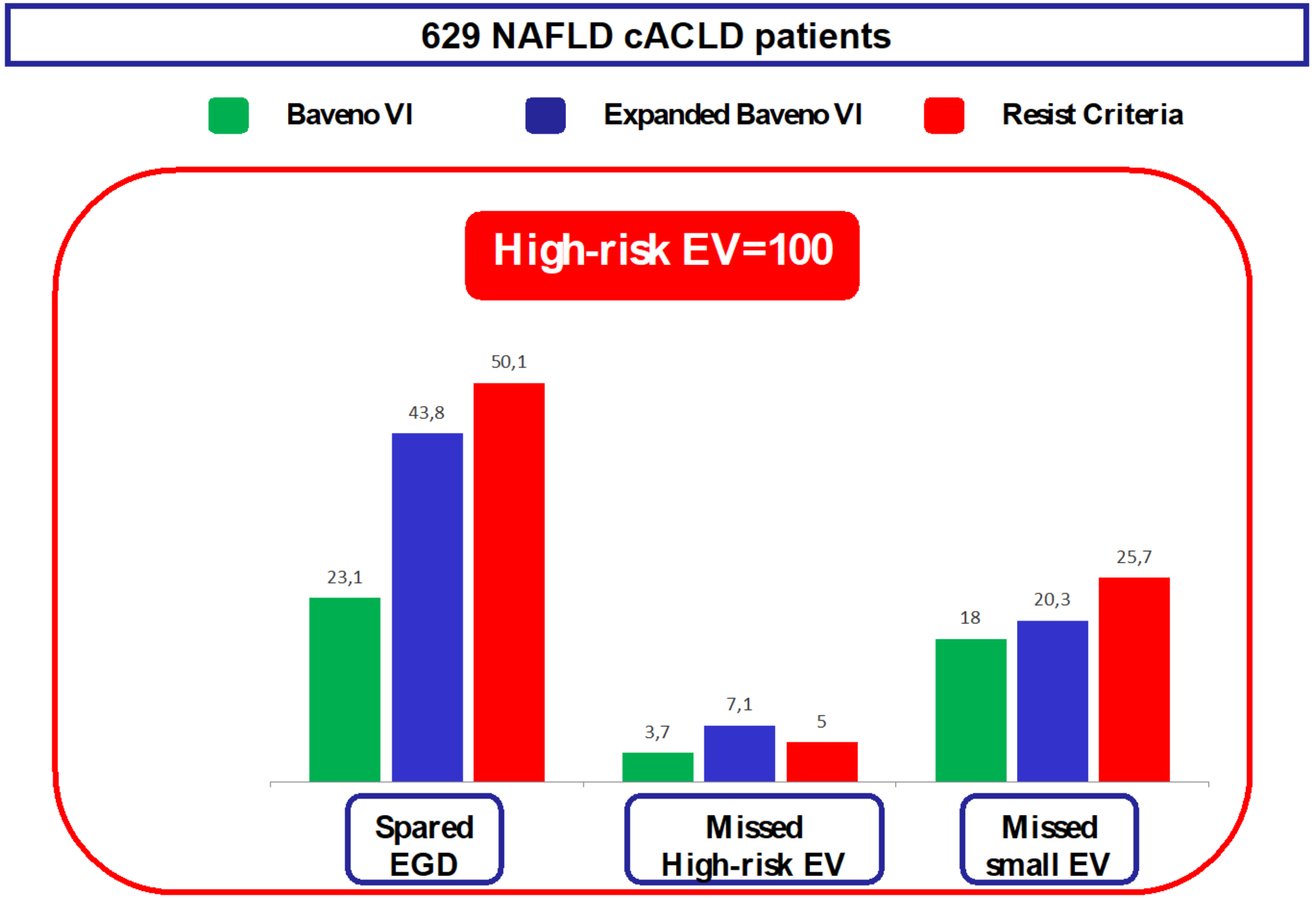
629 NAFLD cACLD patients
Baveno VI
Expanded Baveno VI
Resist Criteria
High-risk EV=100
23,1
43,8
50,1
3,7
7,1
5
18
20,3
25,7
Spared EGD
Missed High-risk EV
Missed small EV

**Fig. S1.**

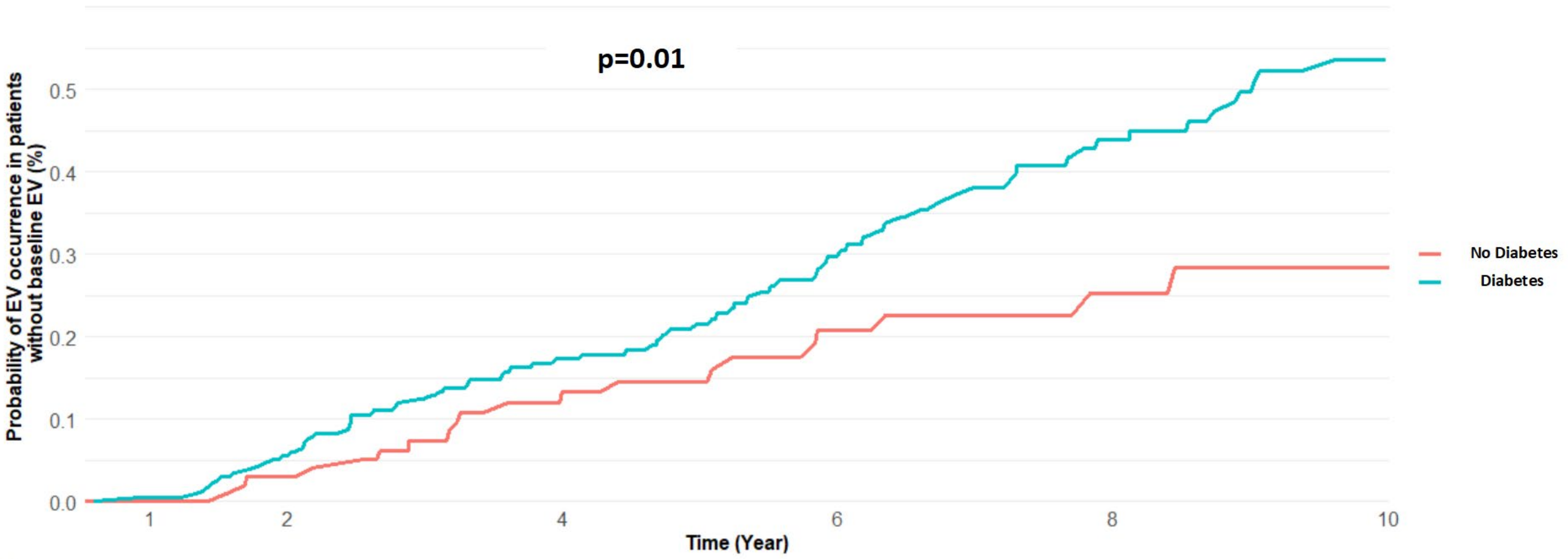


N. at Risk

| | 1 | 2 | 4 | 6 | 8 | 10 |
|---|---|---|---|---|---|---|
| No Diabetes | 102 | 95 | 71 | 48 | 28 | 14 |
| Diabetes | 234 | 215 | 156 | 95 | 55 | 33 |

**Fig. S2A.**

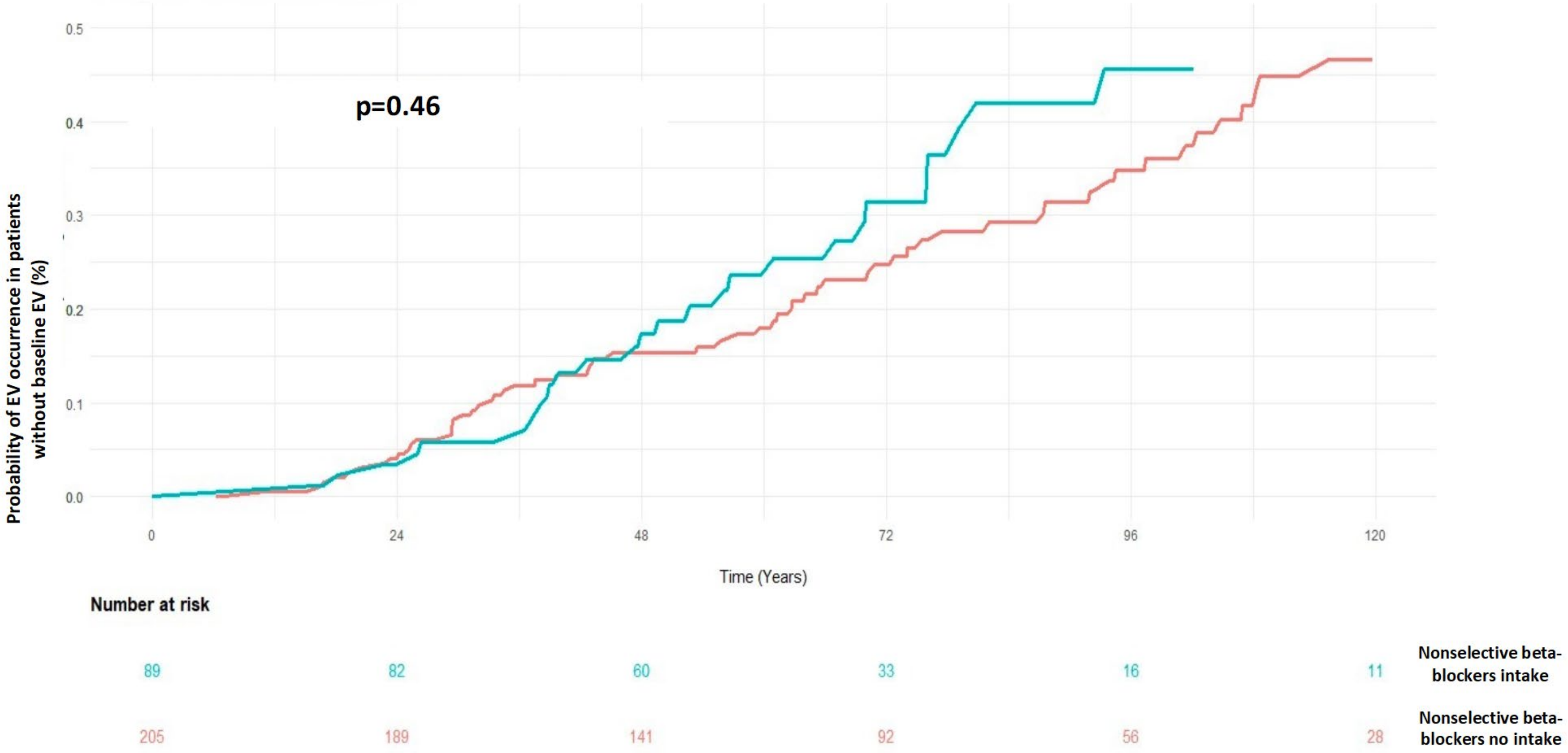
p=0.46
Probability of EV occurrence in patients without baseline EV (%)
0.5
0.4
0.3
0.2
0.1
0.0
0
24
48
72
96
120
Time (Years)
Number at risk
89
82
60
33
16
11
Nonselective beta-blockers intake
205
189
141
92
56
28
Nonselective beta-blockers no intake

**Fig. S2B.**

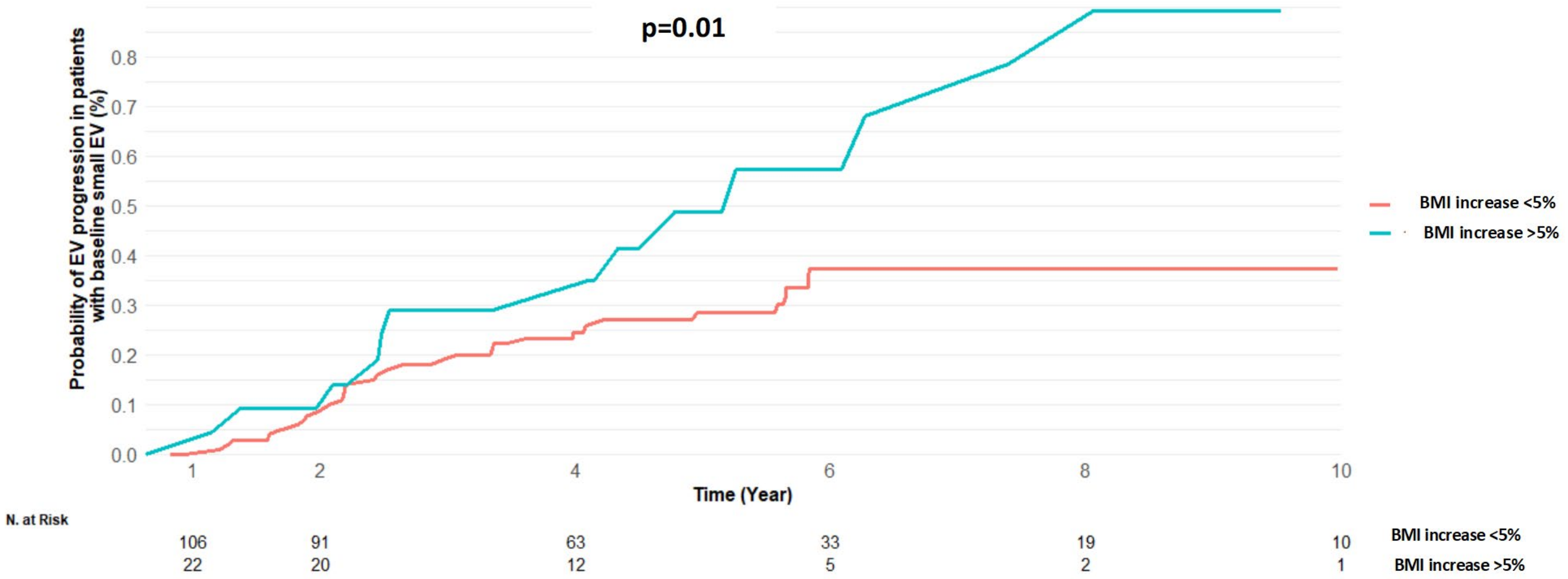
p=0.01
Probability of EV progression in patients with baseline small EV (%)
0.0
0.1
0.2
0.3
0.4
0.5
0.6
0.7
0.8
1
2
4
6
8
10
Time (Year)
BMI increase <5%
BMI increase >5%
N. at Risk
106 91 63 33 19 10 BMI increase <5%
22 20 12 5 2 1 BMI increase >5%

**Fig. S3A.**

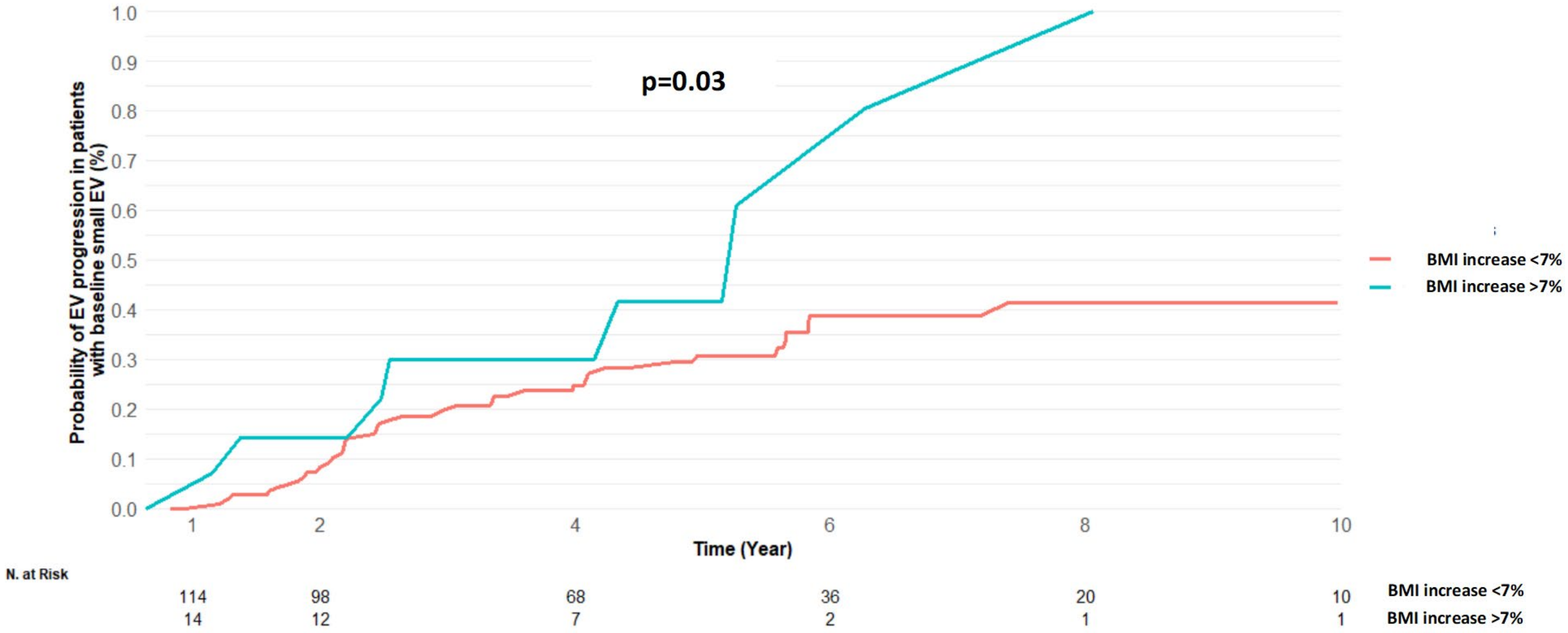
p=0.03
Probability of EV progression in patients with baseline small EV (%)
1.0
0.9
0.8
0.7
0.6
0.5
0.4
0.3
0.2
0.1
0.0
1
2
4
6
8
10
Time (Year)
BMI increase <7%
BMI increase >7%
N. at Risk
114 98 68 36 20 10 BMI increase <7%
14 12 7 2 1 1 BMI increase >7%

Fig. S3B.

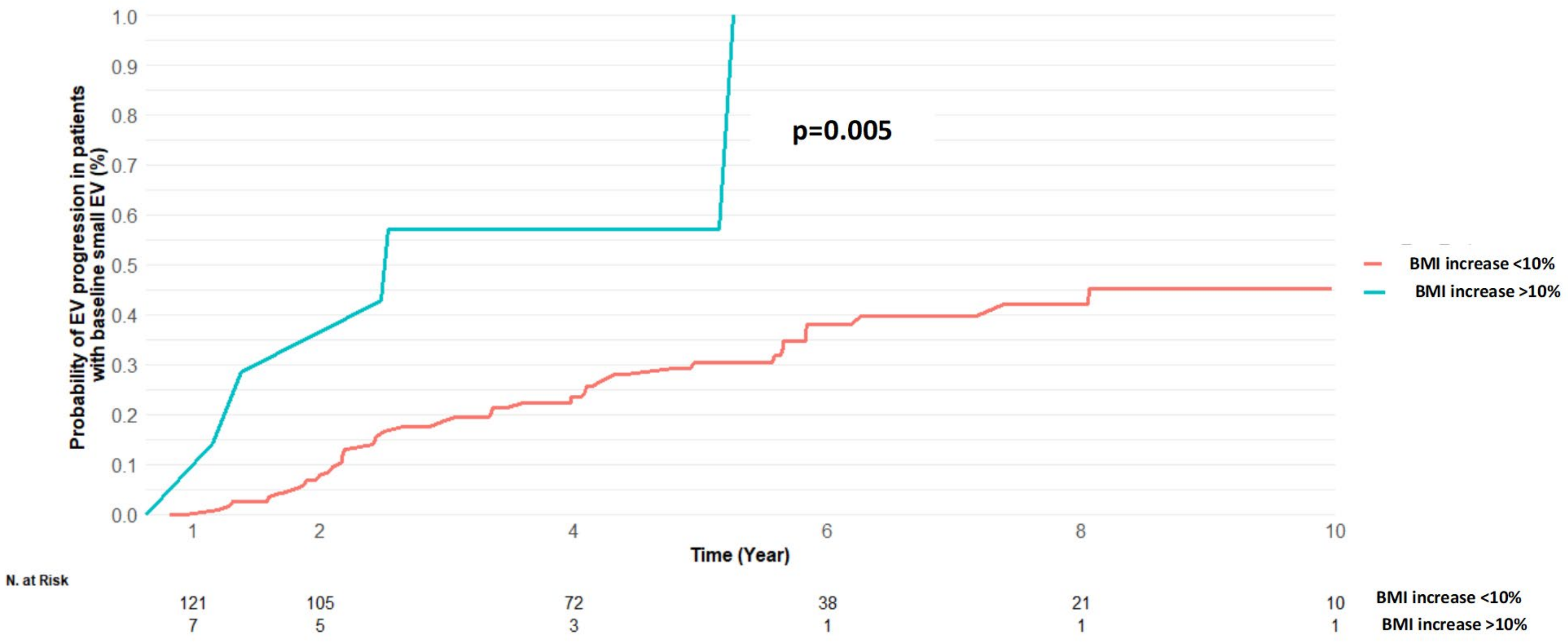

1.0
0.9
0.8
0.7
0.6
0.5
0.4
0.3
0.2
0.1
0.0
Probability of EV progression in patients with baseline small EV (%)
p=0.005
BMI increase <10%
BMI increase >10%
1
2
4
6
8
10
Time (Year)
N. at Risk
121 105 72 38 21 10 BMI increase <10%
7 5 3 1 1 1 BMI increase >10%


Fig. S3C.

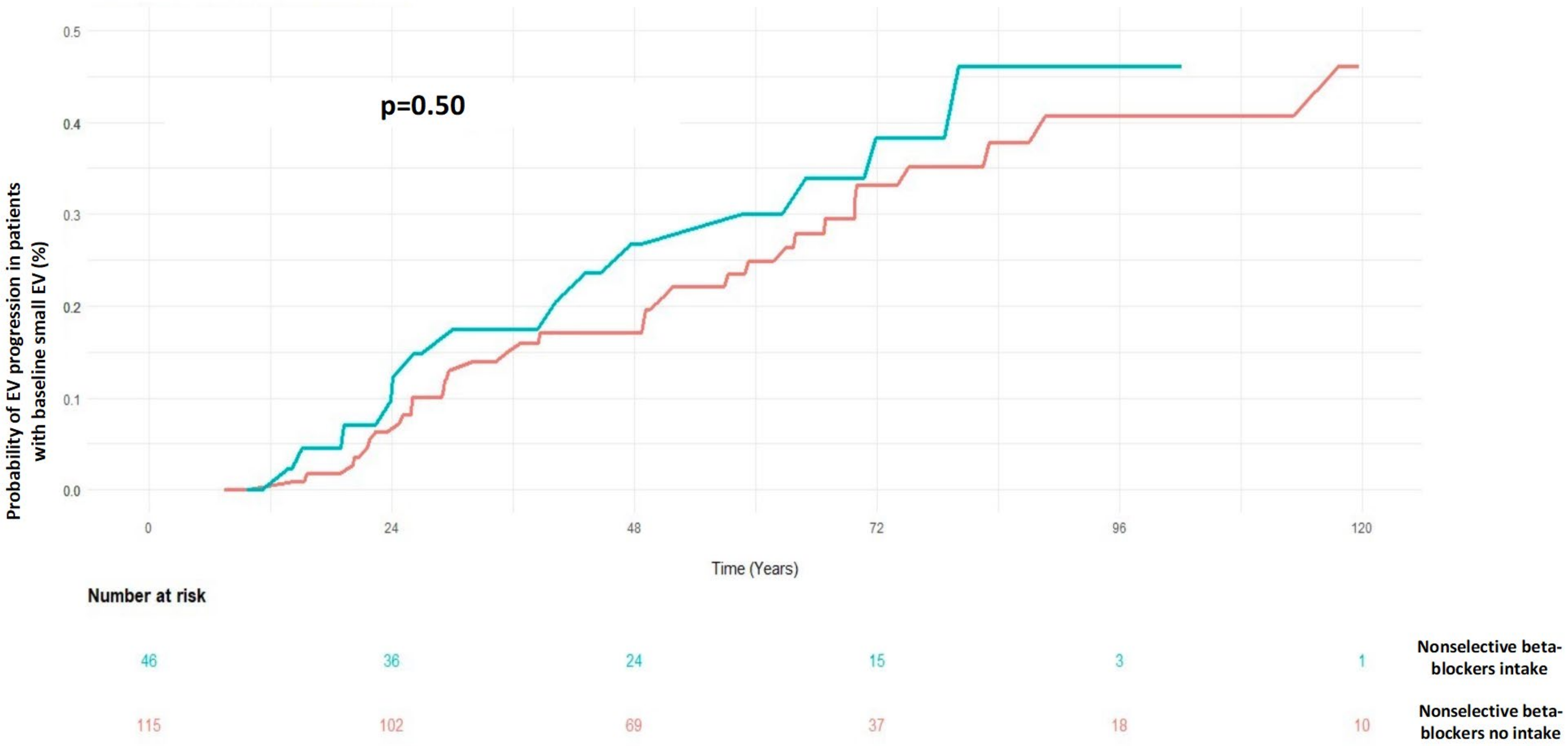
p=0.50
0.5
0.4
0.3
0.2
0.1
0.0
Probability of EV progression in patients with baseline small EV (%)
0
24
48
72
96
120
Time (Years)
Number at risk
46
36
24
15
3
1
Nonselective beta-blockers intake
115
102
69
37
18
10
Nonselective beta-blockers no intake

**Fig. S3D.**

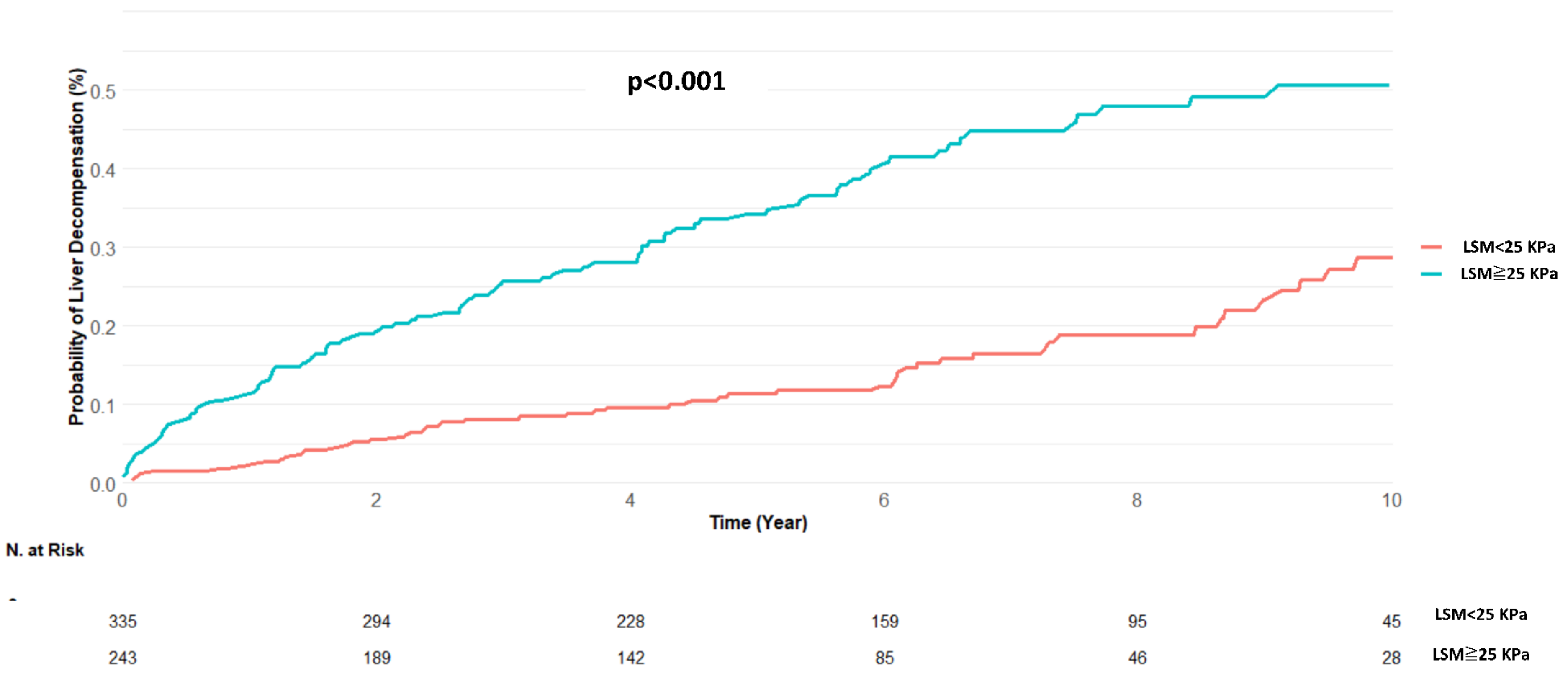
p<0.001
Probability of Liver Decompensation (%)
0.0
0.1
0.2
0.3
0.4
0.5
0
2
4
6
8
10
Time (Year)
LSM<25 KPa
LSM≧25 KPa
N. at Risk
335
294
228
159
95
45
LSM<25 KPa
243
189
142
85
46
28
LSM≧25 KPa

**Fig. S4A.**

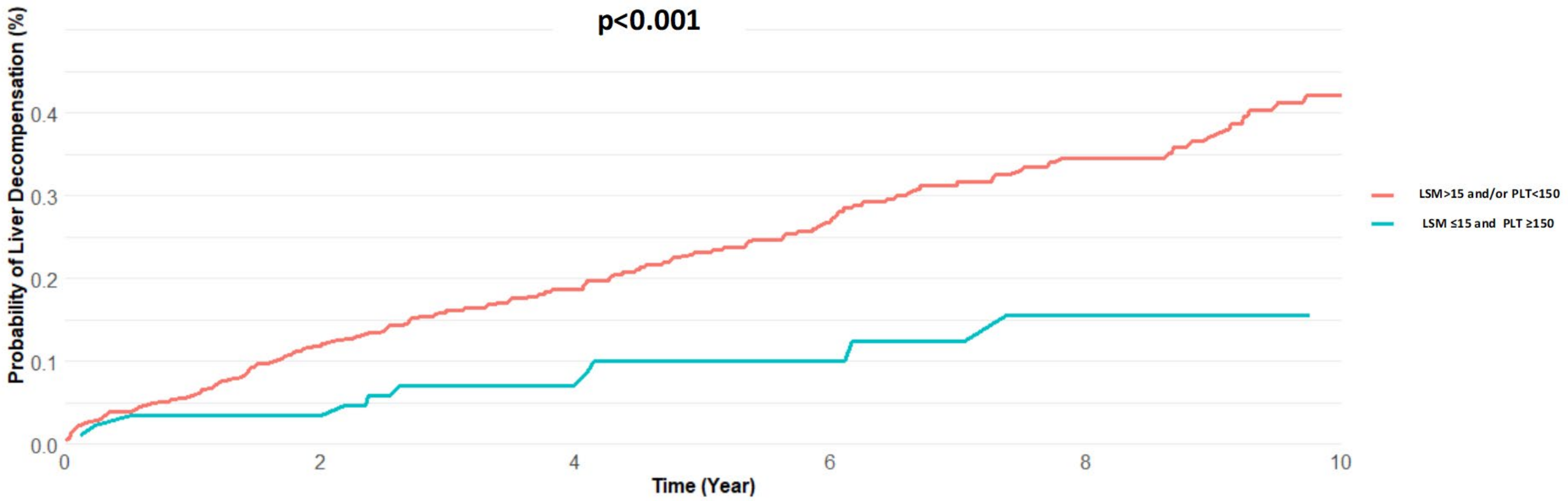


N. at Risk

| | | | | | | |
|---|---|---|---|---|---|---|
| 486 | 404 | 309 | 209 | 122 | 59 | LSM>15 and/or PLT<150 |
| 88 | 82 | 66 | 40 | 25 | 17 | LSM ≤15 and PLT ≥150 |

**Fig. S4B.**

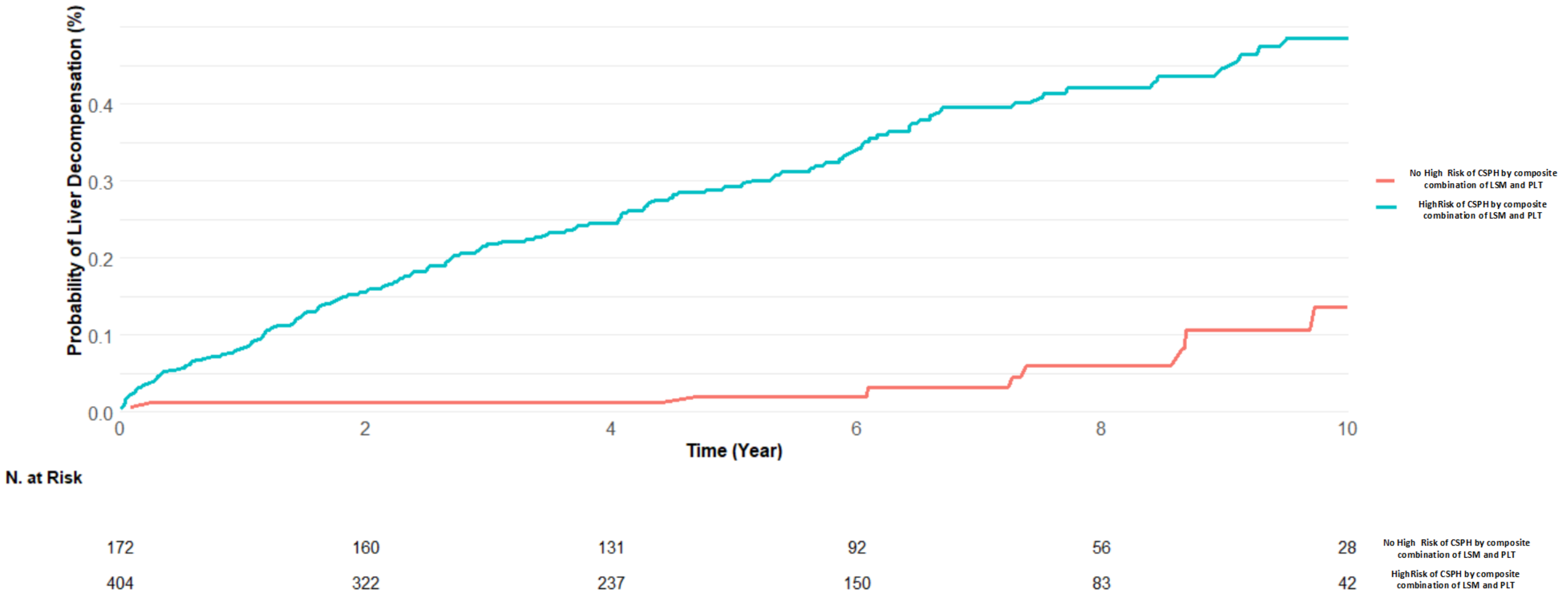
Probability of Liver Decompensation (%)
0.0
0.1
0.2
0.3
0.4
0
2
4
6
8
10
Time (Year)
No High Risk of CSPH by composite combination of LSM and PLT
High Risk of CSPH by composite combination of LSM and PLT
N. at Risk
172 160 131 92 56 28 No High Risk of CSPH by composite combination of LSM and PLT
404 322 237 150 83 42 High Risk of CSPH by composite combination of LSM and PLT

**Fig. S4C.**

**Table S1. Diagnostic Accuracy for High-risk EV of Baveno VI, Extended Baveno VI and Resist Criteria in Patients with NAFLD-related cACLD.**

**Entire Cohort (N=629)**

| **High-risk EV (%)→** | | **100 (15.9)** |
|---|---|---|
| **Baveno VI Criteria**<br>**PLT> 150 X10³ and LSM < 20 kPa**<br>**N=575 (94 High-risk EV)** | Spared Endoscopies (%)<br>Missed High-risk EV n. (%)<br>Missed small EV (%)<br>Sensitivity<br>Specificity<br>NPV<br>PPV | 133(23.1)<br>5(3.7)<br>24 (18.0)<br>94.6%<br>26.6%<br>96.2%<br>20.1% |
| **Extended Baveno VI Criteria**<br>**PLT> 110 X10³ and LSM < 25 kPa**<br>**N=575 (94 High-risk EV)** | Spared Endoscopies (%)<br>Missed High-risk EV n. (%)<br>Missed small EV (%)<br>Sensitivity<br>Specificity<br>NPV<br>PPV | 251(43.8)<br>18(7.1)<br>51 (20.3)<br>80.8%<br>48.4%<br>92.8%<br>23.4% |
| **Resist Criteria**<br>**PLT> 120 X10³ and Albumin < 3.6 g/L**<br>**N=590 (92 High-risk EV)** | Spared Endoscopies (%)<br>Missed High-risk EV n. (%)<br>Missed small EV (%)<br>Sensitivity<br>Specificity<br>NPV<br>PPV | 296(50.1)<br>15(5.0)<br>76(25.7)<br>83.7%<br>56.4%<br>94.9%<br>26.1% |

**Sub-group of Patients where all criteria were concomitantly available (N=547)**

| **High-risk EV (%)→** | | **88 (16.1)** |
|---|---|---|
| **Baveno VI Criteria**<br>**PLT> 150 X10³ and LSM < 20 kPa**<br>**N=547 (88 High-risk EV)** | Spared Endoscopies (%)<br>Missed High-risk EV n. (%)<br>Missed small EV (%)<br>Sensitivity<br>Specificity<br>NPV<br>PPV | 125(22.8)<br>5(4)<br>22(17.6)<br>94.3%<br>26.1%<br>96%<br>19.6% |

| | | |
|---|---|---|
| **Extended Baveno VI Criteria**<br>**PLT> 110 X10$^3$ and LSM < 25 kPa**<br>**N=547 (88 High-risk EV)** | Spared Endoscopies (%)<br>Missed High-risk EV n. (%)<br>Missed small EV (%)<br>Sensitivity<br>Specificity<br>NPV<br>PPV | 234(42.8)<br>15(6.4)<br>49 (20.9)<br>82.9%<br>47.7%<br>93.5%<br>23.3% |
| **Resist Criteria**<br>**PLT> 120 X10$^3$ and Albumin < 3.6 g/L**<br>**N=547 (88 High-risk EV)** | Spared Endoscopies (%)<br>Missed High-risk EV n. (%)<br>Missed small EV (%)<br>Sensitivity<br>Specificity<br>NPV<br>PPV | 285(52.1)<br>14(4.9)<br>72(25.2)<br>84.1%<br>59.1%<br>95.1%<br>28.2% |

**Table S2. Diagnostic accuracy, generated by univariate and multivariate Cox regression models, of baseline EV status, or noninvasive markers of PH for the prediction of liver decompensation in patients with NAFLD-related cACLD.**

| Time | Unadjusted AUC LSM>=25kPa | Unadjusted AUC EV Status | Unadjusted AUC LSM ≤15 kPa plus PLT ≥150x109/L | Unadjusted AUC LSM>25 kPa or LSM between 20-25 kPa plus PLT <150x$10^9$/L, or LSM between 15-20 kPa plus PLT <110x$10^9$/L | Unadjusted ANTICIPATE NASH AUC | Unadjusted AUC 3P ML Model | P value[1] | P value[2] | P value[3] | P value[4] | P value[5] | P value[6] | P value[7] | P value[8] | P value[9] | P value[10] | P value[11] | P value[12] | P value[13] | P value[14] | P value[15] |
|---|---|---|---|---|---|---|---|---|---|---|---|---|---|---|---|---|---|---|---|---|---|
| 1 year | 0.689 | 0.761 | 0.527 | 0.626 | 0.788 | 0.696 | 0.54 | 0.008 | 0.35 | 0.01 | 0.90 | <0.001 | 0.007 | 0.90 | 0.90 | 0.01 | <0.001 | 0.002 | 0.001 | 0.70 | 0.13 |
| 3 years | 0.646 | 0.771 | 0.548 | 0.664 | 0.810 | 0.778 | 0.009 | 0.01 | 0.85 | <0.001 | 0.009 | <0.001 | 0.002 | 0.76 | 0.85 | <0.001 | <0.001 | <0.001 | <0.001 | <0.001 | 0.66 |
| 5 years | 0.648 | 0.720 | 0.549 | 0.682 | 0.795 | 0.781 | 0.26 | 0.01 | 0.43 | <0.001 | 0.005 | <0.001 | 0.47 | 0.19 | 0.31 | <0.011 | <0.001 | <0.001 | <0.001 | 0.003 | 0.61 |
| 8 years | 0.658 | 0.714 | 0.547 | 0.683 | 0.797 | 0.789 | 0.56 | 0.01 | 0.77 | <0.001 | 0.008 | <0.001 | 0.77 | 0.17 | 0.24 | <0.001 | <0.001 | <0.001 | <0.001 | 0.001 | 0.77 |
| | | | | | | | | | | | | | | | | | | | | | |
| | AUC of the model* including LSM>=25kPa | AUC of the model° including EV Status | AUC of the model§ including LSM ≤15 kPa plus PLT ≥150x109/L | AUC of the model& including LSM>25 kPa or LSM between 20-25 kPa plus PLT <150x$10^9$/L, or LSM between 15-20 kPa plus PLT <110x$10^9$/L | AUC of the model$ including ANTICIPATE NASH | AUC of the model% including AUC 3P ML Model | P value[1] | P value[2] | P value[3] | P value[4] | P value[5] | P value[6] | P value[7] | P value[8] | P value[9] | P value[10] | P value[11] | P value[12] | P value[13] | P value[14] | P value[15] |
| 1 year | 0.816 | 0.820 | 0.799 | 0.806 | 0.844 | 0.813 | 0.83 | 0.83 | 0.83 | 0.83 | 0.83 | 0.83 | 0.83 | 0.83 | 0.83 | 0.83 | 0.81 | 0.83 | 0.83 | 0.83 | 0.83 |
| 3 years | 0.882 | 0.890 | 0.854 | 0.873 | 0.900 | 0.894 | 0.84 | 0.61 | 0.94 | 0.61 | 0.42 | 0.57 | 0.74 | 0.91 | 0.57 | 0.65 | 0.33 | 0.34 | 0.59 | 0.50 | 0.94 |
| 5 years | 0.856 | 0.866 | 0.822 | 0.855 | 0.867 | 0.868 | 0.83 | 0.64 | 0.83 | 0.83 | 0.83 | 0.42 | 0.83 | 0.83 | 0.83 | 0.46 | 0.54 | 0.27 | 0.83 | 0.83 | 0.83 |
| 8 years | 0.880 | 0.885 | 0.826 | 0.863 | 0.882 | 0.889 | 0.92 | 0.28 | 0.92 | 0.92 | 0.92 | 0.13 | 0.92 | 0.92 | 0.92 | 0.42 | 0.29 | 0.11 | 0.92 | 0.92 | 0.92 |

**[1]Comparison between EV status and LSM>=25 kPa; [2]Comparison between LSM>=25 kPa and LSM ≤15 kPa plus PLT ≥150x109/L; [3] Comparison between LSM>=25 kPa and LSM>25 kPa or LSM between 20-25 kPa plus PLT <150x$10^9$/L, or LSM between 15-20 kPa plus PLT <110x$10^9$/L; [4] Comparison between LSM>=25 kPa and ANTICIPATE NASH score; [5] Comparison between LSM>=25 kPa and 3P ML model; [6] Comparison between EV status and LSM ≤15 kPa plus PLT ≥150x109/L; [7] Comparison between EV status and LSM>25 kPa or LSM between 20-25 kPa plus PLT <150x$10^9$/L, or LSM between 15-20 kPa plus PLT <110x$10^9$/L; [8]Comparison**

**between EV status and ANTICIPATE NASH score; [9]Comparison between EV status and 3P ML model; [10]Comparison between LSM ≤15 kPa plus PLT ≥150x109/L and LSM>25 kPa or LSM between 20-25 kPa plus PLT <150x109/L, or LSM between 15-20 kPa plus PLT <110x$10^9$/L; [11]Comparison between LSM ≤15 kPa plus PLT ≥150x109/L and ANTICIPATE NASH score; [12]Comparison between LSM ≤15 kPa plus PLT ≥150x109/L and 3P ML model; [13]Comparison between LSM>25 kPa or LSM between 20-25 kPa plus PLT <150x$10^9$/L, or LSM between 15-20 kPa plus PLT <110x$10^9$/L and ANTICIPATE NASH score; [14]Comparison between LSM>25 kPa or LSM between 20-25 kPa plus PLT <150x$10^9$/L, or LSM between 15-20 kPa plus PLT <110x$10^9$/L and 3P ML model; [15]Comparison between ANTICIPATE NASH score and 3P ML model.**

**[*]Variables included in the model: Gender, Age >=65 years, BMI >= 30 Kg/m2, LSM>= 25`kPa, Albumin <3.6 g/dl, Platelet <150x$10^9$/L, statin treatment, nonselective beta-blockers treatment.**

**[°]Variables included in the model: Gender, Age >=65 years, BMI >= 30 Kg/m2, Albumin <3.6 g/dl, Platelet <150x$10^9$/L, EV status, statin treatment, nonselective beta-blockers treatment.**

**[§]Variables included in the model: Gender, Age >=65 years, BMI >= 30 Kg/m2, Albumin <3.6 g/dl, LSM ≤15 kPa plus PLT ≥150x109/L, statin treatment, nonselective beta-blockers treatment.**

**[&]Variables included in the model: Gender, Age >=65 years, BMI >= 30 Kg/m2, Albumin <3.6 g/dl, LSM>25 kPa or LSM between 20-25 kPa plus PLT <150x$10^9$/L, or LSM between 15-20 kPa plus PLT <110x$10^9$/L, statin treatment, nonselective beta-blockers treatment.**

**[$]Variables included in the model: Gender, Age >=65 years, Albumin <3.6 g/dl, ANTICIPATE NASH score, statin treatment, nonselective beta-blockers treatment.**

**[%] Variables included in the model: Gender, Age >=65 years, Albumin <3.6 g/dl, 3P ML Model >0.663, statin treatment, nonselective beta-blockers treatment.**

Comparison among AUROCs was done by using DeLong test.

**Table S3. Adjusted Hazard Ratio by multivariate Cox regression analyses of small and large EV for the prediction of liver decompensation in sub-groups of NAFLD-related cACLD patients stratified according to noninvasive markers of PH.**

| | HR[!] 95%C.I. p value |
|---|---|
| LSM≥25 kPa | small EV 1.92, 1.04-3.54, p=0.03 |
| | large EV 3.11, 1.57-6.18, p=0.001 |
| LSM<25 kPa | small EV 1.79, 0.83-3.85, p=0.13 |
| | large EV 3.74, 1.41-9.92, p=0.008 |
| | |
| LSM ≤15 kPa plus PLT ≥150x109/L | small EV 14.5, 2.07-102.5, p=0.07 |
| | No cases |
| LSM>15 kPa and/or PLT<150x109/L | small EV 2.14, 1.36-3.36, p<0.01 |
| | large EV 3.33, 1.89-5.86, p<0.001 |
| | |
| LSM>25 kPa or LSM between 20-25 kPa plus PLT <150x109/L, or LSM between 15-20 kPa plus PLT <110x109/L | small EV 3.59, 0.55-23.5, p=0.18 |
| | large EV 0.05, 0-3.10, p=0.15 |
| LSM between 20-25 kPa plus PLT ≥150x109/L, or LSM between 15-20 kPa plus PLT ≥110x109/L or LSM<15 kPa | small EV 2.16, 1.33-3.48, p=0.001 |
| | large EV 3.46, 1.99-6.03, p<0.001 |

**Table S4. Actuarial rate of liver decompensation according to follow-up changes in EV, or in noninvasive markers of PH in patients with NAFLD-related cACLD.**

| Time | No baseline, no follow-up EV | Baseline small EV, no follow-up EV | Baseline and follow-up small EV | No baseline EV, follow-up small EV | Progression to large follow-up EV | Baseline large EV |
|---|---|---|---|---|---|---|
| 1 year | 0.8% | 4.2% | 4.8% | 5% | 3.6% | 23.3% |
| 3 years | 1.3% | 9% | 19.5% | 7.5% | 24.6% | 46.4% |
| 5 years | 4.4% | 14.3% | 28.9% | 14.1% | 35.5% | 50.7% |
| 8 years | 8.3% | 14.3% | 42% | 21.4% | 53.9% | 69.4% |
| | | | | | | |
| Time | Baseline LSM<25 kPa, follow-up LSM<25 kPa | Baseline LSM<25 kPa, follow-up LSM>=25 kPa | Baseline LSM>=25 kPa, follow-up LSM<25 kPa | Baseline LSM>=25 kPa, follow-up LSM>=25 kPa | | |
| 1 year | 2.6% | 0% | 4.5% | 13.2% | | |
| 3 years | 6.7% | 0% | 11.2% | 28.3% | | |
| 5 years | 9% | 2.2% | 13.4% | 34.6% | | |
| 8 years | 15.7% | 13.5% | 16% | 55.2% | | |
| | | | | | | |
| Time | Baseline and follow-up LSM ≤15 kPa plus PLT ≥150x10$^9$/L | Baseline LSM ≤15 kPa plus PLT ≥150x10$^9$/L, follow-up LSM>15 kPa and/or PLT<150x10$^9$/L | Baseline LSM>15 kPa and/or PLT<150x10$^9$/L, follow-up LSM ≤15 kPa plus PLT ≥150x10$^9$/L | Baseline and follow-up LSM>15 kPa and/or PLT<150x10$^9$/L | | |
| 1 year | 0% | 8.6% | 2% | 2.6% | | |
| 3 years | 0% | 14.5% | 2% | 11.8% | | |
| 5 years | 0% | 17.5% | 5.2% | 20% | | |
| 8 years | 6.7% | 21.9% | 18.7% | 30.5% | | |
| | | | | | | |
| Time | Baseline and follow-up LSM between 20-25 kPa plus PLT >150x10$^9$/L, or | Baseline LSM between 20-25 kPa plus PLT >150x10$^9$/L, or LSM | Baseline LSM>25 kPa or LSM between 20-25 kPa plus PLT <150x10$^9$/L, or | Baseline and follow-up LSM>25 kPa or LSM between 20-25 kPa plus | | |

| | | | | | | |
|---|---|---|---|---|---|---|
| | LSM between 15-20 kPa plus PLT >110x10$^9$/L or LSM<15 kPa | between 15-20 kPa plus PLT >110x10$^9$/L or LSM<15 kPa<br>Follow-up LSM>25 kPa or LSM between 20-25 kPa plus PLT <150x10$^9$/L, or LSM between 15-20 kPa plus PLT <110x10$^9$/L | LSM between 15-20 kPa plus PLT <110x10$^9$/L<br>Follow-up LSM between 20-25 kPa plus PLT >150x10$^9$/L, or LSM between 15-20 kPa plus PLT >110x10$^9$/L or LSM<15 kPa | PLT <150x10$^9$/L, or LSM between 15-20 kPa plus PLT <110x10$^9$/L | | |
| 1 year | 1.8% | 0% | 4% | 8% | | |
| 3 years | 1.8% | 0% | 8.1% | 19.8% | | |
| 5 years | 1.8% | 2.9% | 8.1% | 25.5% | | |
| 8 years | 6.5% | 6.6% | 8.1% | 41.5% | | |

**Kaplan Meier curves were also used to depict the time-dependent risk of developing events.**

**Table S5. Diagnostic accuracy, generated by univariate and multivariate Cox regression models, of changes during follow-up of EV status, or of noninvasive markers of PH for the prediction of LD in patients with NAFLD-related cACLD.**

| Time | Unadjusted AUC LSM>=25kPa changes | Unadjusted AUC EV Status changes | Unadjusted AUC LSM ≤15 kPa plus PLT ≥150x10⁹/L changes | Unadjusted AUC LSM>25 kPa or LSM between 20-25 kPa plus PLT <150x10⁹/L, or LSM between 15-20 kPa plus PLT <110x10⁹/L changes | P value[1] | P value[2] | P value[3] | P value[4] | P value[5] | P value[6] |
|---|---|---|---|---|---|---|---|---|---|---|
| 1 year | 0.621 | 0.732 | 0.547 | 0.601 | 0.19 | 0.36 | 0.62 | <0.001 | 0.01 | 0.46 |
| 3 years | 0.607 | 0.766 | 0.548 | 0.617 | <0.001 | 0.16 | 0.70 | <0.001 | <0.001 | 0.02 |
| 5 years | 0.632 | 0.716 | 0.545 | 0.649 | 0.02 | 0.01 | 0.47 | <0.001 | 0.02 | <0.001 |
| 8 years | 0.634 | 0.751 | 0.521 | 0.658 | 0.001 | 0.001 | 0.36 | <0.001 | 0.002 | <0.001 |
| | | | | | | | | | | |
| | AUC of the model[*] including LSM>=25kPa changes | AUC of the model[°] including EV Status changes | AUC of the model[§] including LSM ≤15 kPa plus PLT ≥150x10⁹/L changes | AUC of the model[&] including LSM>25 kPa or LSM between 20-25 kPa plus PLT <150x10⁹/L, or LSM between 15-20 kPa plus PLT <110x109/L changes | P value[1] | P value[2] | P value[3] | P value[4] | P value[5] | P value[6] |
| 1 year | 0.786 | 0.800 | 0.800 | 0.805 | 0.99 | 0.99 | 0.99 | 0.99 | 0.99 | 0.99 |
| 3 years | 0.844 | 0.868 | 0.839 | 0.840 | 0.41 | 0.94 | 0.94 | 0.27 | 0.27 | 0.94 |
| 5 years | 0.832 | 0.827 | 0.793 | 0.826 | 0.96 | 0.27 | 0.96 | 0.20 | 0.96 | 0.07 |
| 8 years | 0.841 | 0.839 | 0.770 | 0.822 | 0.92 | 0.01 | 0.67 | 0.008 | 0.78 | 0.01 |

**[1]Comparison between changes in EV status and LSM>=25 kPa; [2]Comparison between changes in LSM>=25 kPa and LSM ≤15 kPa plus PLT ≥150x109/L; [3] Comparison between changes in LSM>=25 kPa and LSM>25 kPa or LSM between 20-25 kPa plus PLT <150x10⁹/L, or LSM between 15-20 kPa plus PLT <110x10⁹/L; [4] Comparison between changes in EV status and LSM ≤15 kPa plus PLT ≥150x109/L; [5] Comparison between changes in EV status and LSM>25 kPa or LSM between 20-25 kPa plus PLT <150x10⁹/L, or LSM between 15-20 kPa plus PLT <110x10⁹/L; [6]Comparison between changes in LSM ≤15 kPa plus PLT ≥150x109/L and LSM>25 kPa or LSM between 20-25 kPa plus PLT <150x10⁹/L, or LSM between 15-20 kPa plus PLT <110x10⁹/L.**

**[*]Variables included in the model: Gender, Age >=65 years, BMI >= 30 Kg/m2, changes in LSM>= 25`kPa, Albumin <3.6 g/dl, Platelet <150x10⁹/L, statin treatment, nonselective beta-blockers treatment.**

**°Variables included in the model: Gender, Age >=65 years, BMI >= 30 Kg/m2, Albumin <3.6 g/dl, Platelet <150x$10^9$/L, changes in EV status, statin treatment, nonselective beta-blockers treatment.**

**§Variables included in the model: Gender, Age >=65 years, BMI >= 30 Kg/m2, Albumin <3.6 g/dl, changes in LSM ≤15 kPa plus PLT ≥150x109/L, statin treatment, nonselective beta-blockers treatment.**

**&Variables included in the model: Gender, Age >=65 years, BMI >= 30 Kg/m2, Albumin <3.6 g/dl, changes in LSM>25 kPa or LSM between 20-25 kPa plus PLT <150x$10^9$/L, or LSM between 15-20 kPa plus PLT <110x109/L, statin treatment, nonselective beta-blockers treatment.**

Comparison among AUROCs was done by using DeLong test.
